# Stiffness‐Tunable Neurotentacles for Minimally Invasive Implantation and Long‐Term Neural Activity Recordings

**DOI:** 10.1002/advs.202505100

**Published:** 2025-07-21

**Authors:** Yang Wang, Xing Xu, Xiaowei Yang, Rongyu Tang, Ying Chen, Shan Zang, Yijun Wang, Jing Liang, Weihua Pei

**Affiliations:** ^1^ Laboratory of Solid State Optoelectronics Information Technology Institute of Semiconductors Chinese Academy of Sciences Beijing 100083 China; ^2^ University of Chinese Academy of Sciences Beijing 100049 China; ^3^ State Key Laboratory of Cognitive Science and Mental Health Institute of Psychology Chinese Academy of Sciences Beijing 100101 China; ^4^ Department of Psychology University of Chinese Academy of Sciences Beijing 100049 China; ^5^ Foreign Language Teaching and Research Press Beijing 100089 China

**Keywords:** electrophysiology recording, flexible electrodes, implantation method, neurotentacles, stiffness‐tunable

## Abstract

Flexible microelectrodes are ideal for chronic neural recordings; however, their low bending strength poses challenges during probe insertion. Here, a stiffness‐tunable polyimide probe, termed Neurotentacle, is proposed for deep brain implantation. Its tunability is enabled by embedded microchannels with controllable liquid pressure. During insertion, the Neurotentacle becomes stiff under elevated internal pressure, allowing penetration of brain tissue without additional materials or tools. Once inserted, it regains flexibility by reducing the internal pressure. The novel ultra‐thin microchannel fabrication technique enables the Neurotentacle to maintain dimensions similar to conventional flexible probes. This minimizes tissue damage during insertion while ensuring long‐term biocompatibility and stability, confirmed by histological evaluations in both acute and chronic animal models. In long‐term recordings, Neurotentacles outperform traditional shuttle‐assisted implantation methods. The technique is straightforward, controllable, and does not require complex devices, making it ideal for minimally invasive implantation and long‐term neural recordings.

## Graphical abstract

A hydraulically actuated “Neurotentacle” probe offers tunable stiffness for precise implantation into the deep brain without rigid shuttles or dissolvable coatings. It stiffens during insertion to penetrate tissue and softens afterward to minimize damage and enable stable long‐term recordings. This approach provides a minimally invasive interface for chronic neural monitoring and brain–computer interaction.

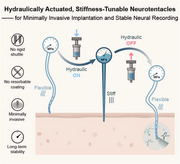

## 1 Introduction

Implantable microelectrodes are capable of acquiring electrophysiological signals of neurons with high temporal‐spatial resolution, which is of great importance in brain‐computer interface research.^[^ ^1^ ^]^ In particular, the flexible neural microelectrodes have been demonstrated to exhibit excellent biocompatibility, which effectively reduces the immune response of brain tissue and extends the in vivo longevity of the device.^[^ ^2^, ^3^, ^4^ ^]^ Flexible electrodes have been fabricated from diverse materials, including polyimide (PI),^[^ ^5^, ^6^, ^7^ ^]^ parylene,^[^ ^8^, ^9^ ^]^ and SU‐8.^[^ ^10^, ^11^ ^]^ However, the low Young's modulus (commonly used to describe the stiffness of materials) of these materials results in insufficient low bending stiffness for most of the flexible probes,^[^ ^12^, ^13^ ^]^ which presents a challenge for their implantation into the brain.

A variety of implantation methods have been developed to facilitate the delivery of flexible electrodes into the brain. These methods can be broadly divided into two categories: methods of reinforcing flexible electrodes with sacrificial materials (RFSM methods) and methods of inserting flexible electrodes with auxiliary tools (IFAT methods).^[^ ^14^ ^]^ The RFSM methods typically strengthen the flexible probes by employing biodegradable materials, including gelatin,^[^ ^15^ ^]^ maltose,^[^ ^16^ ^]^ dextran,^[^ ^17^ ^]^ polyethylene glycol (PEG),^[^ ^3^, ^18^ ^]^ polyacetate,^[^ ^19^ ^]^ silk proteins,^[^ ^20^ ^]^ and some other polymers with similar properties.^[^ ^21^ ^]^ These materials can increase the stiffness of the probes prior to implantation, enabling them to penetrate the brain. Following implantation, the degradation of these materials within the brain allows the probes to recover their flexibility. However, the footprint of the reinforced flexible electrode is usually much larger than the original, enlarging the acute damage caused by the probe insertion. In addition, these materials dissolve rapidly under physiological conditions, making it difficult to manipulate the insertion speed. The IFAT methods temporarily attach the flexible probes to a rigid tool such as a silicon shuttle or metal microneedle and then withdraw the tool after the electrodes are inserted. The attachment can be achieved by self‐adsorption effect,^[^ ^22^ ^]^ water‐soluble adhesive bonding,^[^ ^23^ ^]^ needle‐hole assembling,^[^ ^11^, ^24^, ^25^ ^]^ syringe holding,^[^ ^26^ ^]^ and so on. However, these auxiliary tools are also much larger than the flexible electrodes themselves. Moreover, they could cause secondary damage to brain tissue when withdrawn, as well as disturb or even bring out the inserted flexible electrodes.

In addition, to minimize insertion damage, the optimal implantation should not involve the use of any auxiliary tools or materials that would enlarge the flexible electrodes to an excessive degree. Some innovative insertion methods, such as magnetic actuation^[^ ^27^ ^]^ and microfluidic actuation,^[^ ^28^ ^]^ have the potential to meet this need. Nevertheless, these methods still require optimization in terms of drive force, device complexity, insertion speed, and other aspects. Some strength‐adaptive materials can be integrated with the electrode substrate, such as nanocomposites,^[^ ^29^ ^]^ shape memory polymers,^[^ ^30^ ^]^ and liquid crystal polymers.^[^ ^31^ ^]^ These materials are sufficiently stiff to penetrate brain tissue before insertion and will gradually soften under physiological conditions after insertion. However, such materials typically require complex processing procedures to avoid the conditions that could denature them, such as the high temperatures or some chemical solution environments common in Micro‐Electro‐Mechanical Systems (MEMS) process. Moreover, although these materials can adjust stiffness, the range of adjustment is quite limited when the probe size is very small.

In this study, we designed and prepared a Neurotentacle probe with tunable stiffness based on liquid pressure. The term “Neurotentacle” was inspired by the octopus tentacle, which can dynamically adjust its stiffness through internal pressure modulation, enabling both softness and rigidity on demand. No auxiliary materials or tools other than saline were employed for the insertion of the Neurotentacle into the brain. Inside the Neurotentacle, there is a microchannel, through which the liquid can be injected and the pressure can be regulated to adjust the stiffness of the probe. Based on the developed ultra‐thin microchannel process, the encapsulated Neurotentacles had comparable footprints to the regular flexible probes, as evidenced by previous literature.^[^ ^6^, ^24^, ^25^, ^32^, ^33^ ^]^ There was only a slight expansion of the Neurotentacle volume during insertion, and no further damage was caused by retracting the auxiliary tools, thus greatly reducing the acute damage. In addition, chronically implanted Neurotentacles enabled long‐term stable neural activity recordings in mice with a high yield of spikes and high signal‐to‐noise ratios (SNR).

## 2 Results

### 2.1 Design of Neurotentacles

**Figure 1:**
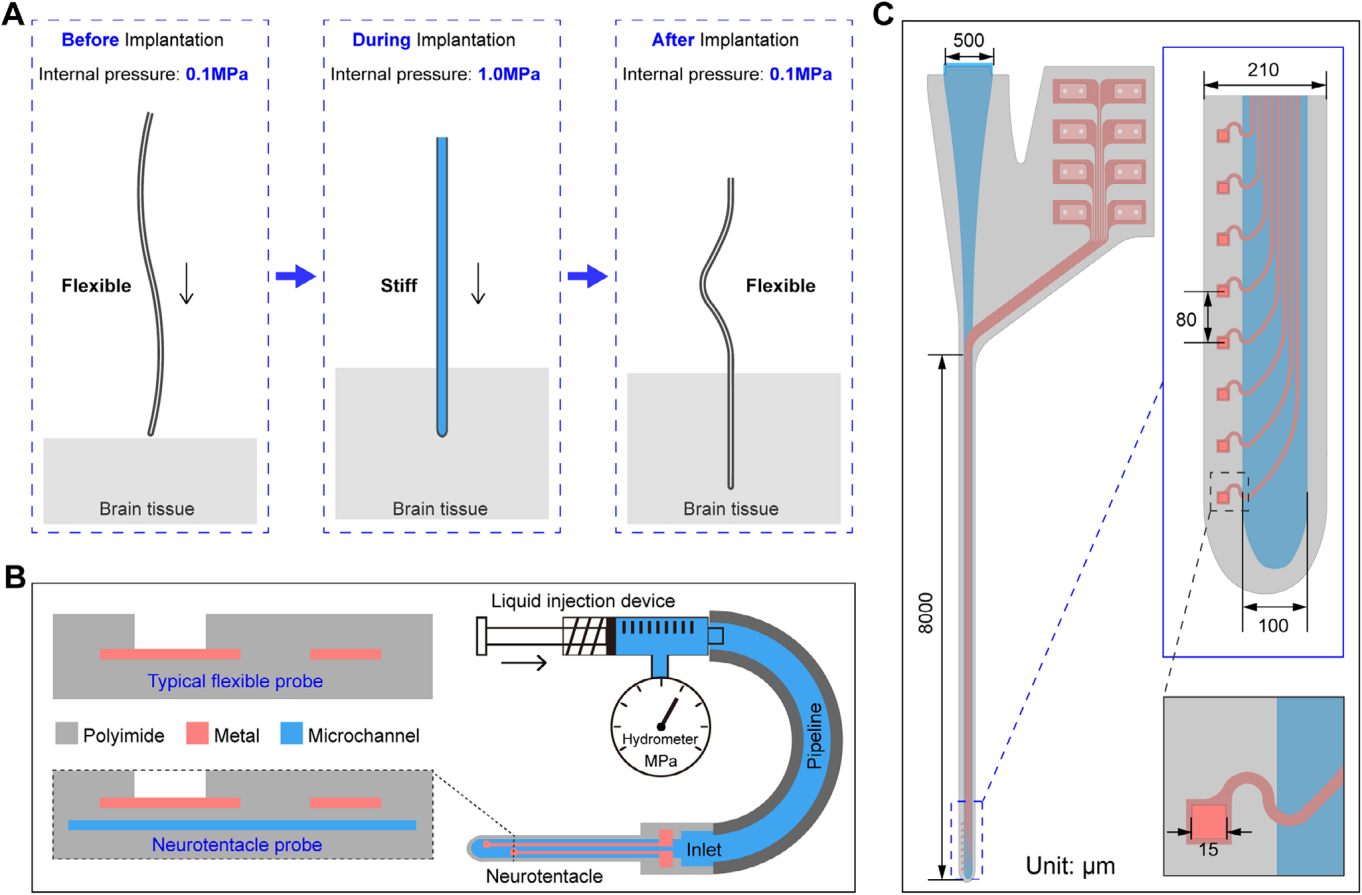
Design of Neurotentacles. A) Implantation process of the Neurotentacle based on variable stiffness. B) Schematic diagram of the overall design and packaging of the Neurotentacle. C) Layout design of the Neurotentacle.

The Neurotentacle probe's mechanical strength varies with internal pressure. This allows it to increase the strength to penetrate brain tissue before implantation and decrease the strength to recover flexibility after implantation, as shown in **Figure** **1**A and Movie S1 (Supporting Information). The key to Neurotentacle design is the integration of a microchannel into the typical PI flexible probe. Compared with the common sandwich‐type PI probes (PI‐Metal‐PI),^[^ ^6^, ^24^ ^]^ the Neurotentacle has an additional microchannel in the middle of the insulating layer (PI‐Microchannel‐PI‐Au‐PI), as shown in Figure 1B. At the rear end of the Neurotentacle, there reverses an inlet for the liquid to enter the microchannel. A liquid injection device is connected to the inlet via a pipeline. It can pump liquid into the probe and regulate its internal pressure. A hydrometer is mounted on the injection device for monitoring the liquid pressure in real‐time.

The layout of the Neurotentacle is shown in Figure 1C. The shank is 8 mm long, which is sufficient to cover the entire brain area of small rodents. It has a width of 210 µm, with the microchannel width being 100 µm. At the front end of the probe, there are eight recording sites with a diameter of 15 µm and a center‐to‐center distance of 80 µm. The metal traces are above the microchannel, and all the recording sites are arranged at the edge of the shank. This layout is partly because it is easier to capture neuronal signals at the edges, and partly to avoid over‐etching to break the microchannel. The metal traces are designed to be S‐shaped at the edge of the microchannel to enhance their tensile resistance, thus better adapting to the variable morphology of the probe during the filling and emptying of the liquid. At the back end of the Neurotentacle, the liquid inlet is separated from the metal pads to prevent potential conflicts between sealing the pipeline and establishing the electrical connection during subsequent operations.

### 2.2 Fabrication of Neurotentacles

**Figure 2:**
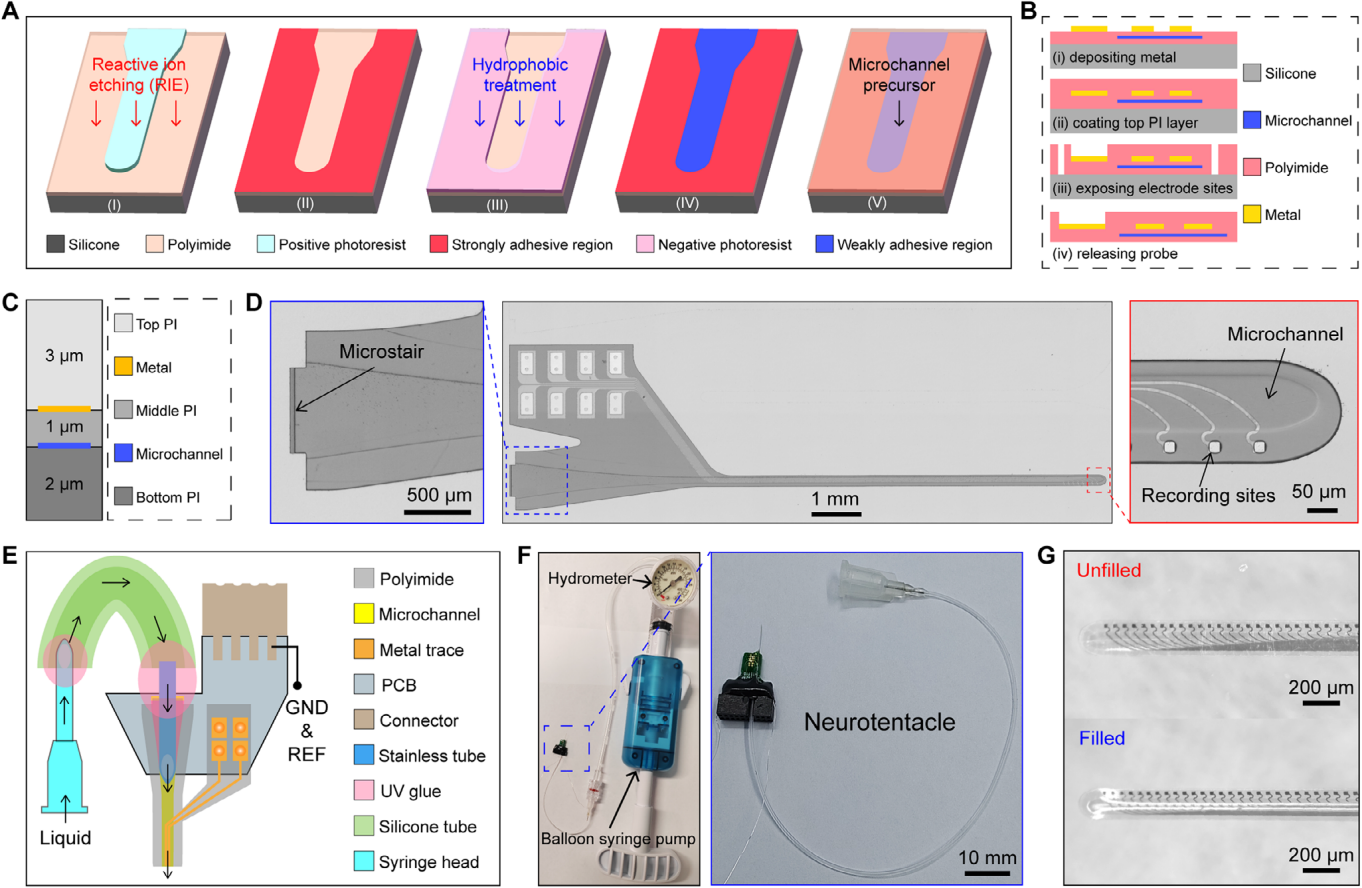
Fabrication and Encapsulation of Neurotentacles. A) Process flow for the preparation of ultra‐thin microchannel precursor. B) Procedures for fabricating the flexible probe after constructing the microchannel precursor. C) Optimized thickness distribution of the three PI layers in the Neurotentacle: bottom layer 2 µm, middle layer 1 µm, and top layer 3 µm. D) A prepared Neurotentacle probe based on the MEMS process (Middle). Liquid inlet at the end of the probe (Left). The tip of the probe with visible microchannel and recording sites(Right). E) Schematic diagram of encapsulating the Neurotentacle with the liquid pathway and electrical connector. F) A physically encapsulated Neurotentacle and a syringe pump for applying the hydraulic pressure. G) Comparison of the morphology of a Neurotentacle under the filled and unfilled state.

The preparation of the microchannel is the most important part of fabricating the Neurotentacle. It is related to many aspects such as fabrication difficulty, mechanical strength of the microchannel, probe thickness, and encapsulation difficulty. Here, we proposed an innovative process flow for fabricating the ultra‐thin microchannel based on differences in adhesion, as shown in **Figure** **2**A. Reactive ion etching and hydrophobic treatment were employed to create regions with different adhesions on the first PI layer. When the second PI layer was cured, the strongly adhesive regions bonded together and the weakly adhesive regions formed the microchannel precursor (microchannel that is unopened and non‐functionalized). Once the Neurotentacle is encapsulated, the liquid injected into the microchannel precursor can open it up, thereby forming the microchannel. The microchannel prepared based on this method is characterized by an extremely small volume in the unfilled state, which greatly reduces the overall thickness of the probe. Its preparation process is easy to realize and involves only the materials and equipment commonly used to fabricate PI‐based flexible probes.

After forming the microchannel precursor, the Neurotentacle is fabricated with the same process as a conventional PI probe, as shown in Figure 2B. The presence of the microchannel results in the device containing three layers of PI. The method of thinning the PI membrane^[^ ^34^ ^]^ is employed here to maintain the overall thickness of the Neurotentacle at the same level as that of conventional PI probes. A kind of PI (PI2611) was mixed with NMP (N‐Methyl‐2‐pyrrolidone) in a mass ratio of 4:1 to obtain the diluted PI. By controlling the spin‐coating speed, the PI films can be prepared with different thicknesses from 1 to 2 µm. The relationship between the thickness and the spin‐coating speed is shown in Figure S1 (Supporting Information). It should be noted that the probe thickness is not equally proportional for each layer of PI. The thickness distribution of the top, middle, and bottom PI layers needs to be optimized for two reasons. First, it is necessary to ensure that the PI thicknesses above and below the metal layer are uniform to avoid self‐bending of the released probes (Figure S2, Supporting Information). This means that the total thickness of the middle and bottom PIs should ideally be the same as that of the top PI. Second, the bottom PI should be as thick as possible to avoid breaking the microchannel during encapsulation. The ideal optimized PI thickness distribution is shown in Figure 2C. The prepared Neurotentacle is shown in Figure 2D. The total thickness was ≈6.2 µm, with the top, middle, and bottom PI layers measuring 3.3 µm, 0.9 µm, and 2.0 µm, respectively, closely matching our designed thickness. Such a Neurotentacle is as thin as the typical PI‐based flexible probe.^[^ ^6^, ^25^ ^]^

### 2.3 Encapsulation of Neurotentacles

To facilitate the opening of the microchannel, a “microstair” (stair‐like microstructure) is reserved at the end of the probe (Figure 2D). Along the bottom of the microstair, the PI can be split apart by using a sharp object, thus forming the inlet of the microchannel (Figure S3, Supporting Information). A stainless tube was inserted into the microchannel as the relay. Then a silicone tube and a syringe head were sequentially connected to the microchannel, as shown in Figure 2E. All the joints were sealed with ultraviolet (UV) glue. During the encapsulation of the liquid pathway, the Neurotentacle was also electrically encapsulated with the printed circuit board (PCB) and connector. The detailed encapsulation procedures (Figure S4, Supporting Information) can be found in Materials and Methods.

A fully encapsulated Neurotentacle is shown in Figure 2F. A medical Balloon Syringe Pump was used to inject liquid into the Neurotentacle. It can provide a stable pressure for the Neurotentacle, and the hydrometer on it can monitor the pressure in real time with a maximum range of 3 MPa. The microchannels can be easily braced by the deionized (DI) water from the syringe pump and have a pressure resistance of at least 2 MPa. When the water is pumped out, the Neurotentacles can rapidly return to the membrane shape. As shown in Figure 2G, there is a notable difference between the probe morphology in the unfilled and filled states. These results primarily demonstrate the feasibility of our proposed method for preparing ultra‐thin microchannels as well as the reliability and robustness of the microchannels.

### 2.4 Highly Tunable Stiffness of Neurotentacles with Liquid Pressure

**Figure 3:**
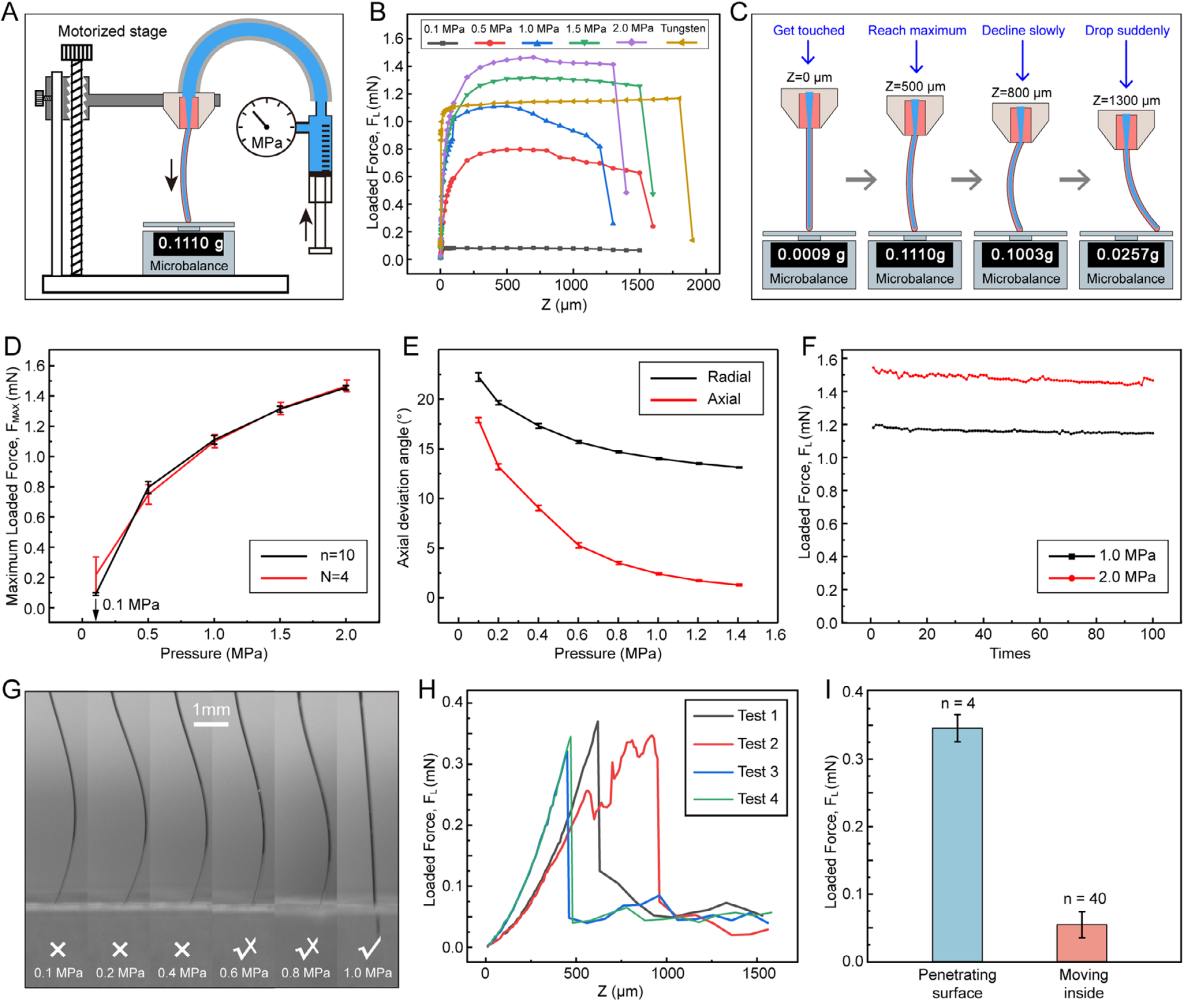
Mechanical performance of Neurotentacles. A) Experimental setup for testing Neurotentacles based on a motorized stage and microbalance. B) The relationship of loaded force (F_L_) and downward displacement (Z) for Neurotentacles under different pressures and the control tungsten wire (linear scale on the x‐axis). C) Corresponding states of the tested Neurotentacles at different stages of the “F_L_‐Z” curve. D) The relationship between average F_MAX_ and pressure for a Neurotentacle (n = 10, mean ± SD) and for different Neurotentacles (N = 4, mean ± SD). E) The relationship between axial deviation angle and pressure for the Neurotentacle under separately applied radial and axial forces (n = 3, mean ± SD). F) Fatigue testing of a Neurotentacle at the pressure of 1 MPa and 2 MPa. G) Testing whether Neurotentacles can penetrate the brain‐like gel at different pressures (0.1–1.0 MPa). H) The “F_L_‐Z” relationships for 1‐MPa Neurotentacles on the brain‐like gel. I) The force required for Neurotentacles to penetrate the gel and to travel inside the gel.

The strength of the Neurotentacle is related to the pressure inside the microchannel. Quantitative tests were conducted to investigate the relationship between the maximum loaded force (F_MAX_) and the liquid pressure of the Neurotentacles. The experimental setup is shown in **Figure** **3**A. The Neurotentacle was fixed to a motorized stage, which can determine the downward displacement (Z). A microbalance was used to test the loaded force (F_L_) on the probe. The quasi‐static relationships of “F_L_‐Z” under different pressures are shown in Figure 3B and Figure S5 (Supporting Information). Each curve represents measurements conducted on the same Neurotentacle probe under different testing conditions. The pressure of 0.1 MPa was used as a control, corresponding to the unfilled state and indicating that the pressure inside and outside the microchannel is equal. A tungsten wire with a diameter of 30 µm and a length of 15 mm was included as a control. Such wires are commonly used as microwire neural electrodes and can reliably penetrate brain tissue without buckling. The origin of displacement (Z = 0) denotes that the probe tip had just touched the microbalance. The results show that the Neurotentacle at all pressures, except for the unfilled state, exhibits similar mechanical properties. The loaded force increased rapidly during initial displacement, reaching 90% of the maximum value within 200 µm (Figure S5, Supporting Information). Afterward, the force increases slowly with displacement until reaching the maximum value (F_MAX_) and then falls smoothly. Eventually, at a certain displacement, the force suddenly drops to a very small value. The different probe states shown in Figure 3C correspond to variations in the horizontal axis variable in Figure 3B. It can be clearly seen that the F_MAX_ of the probe increases substantially compared to the unfilled state (0.1 MPa) and rises apparently as the pressure increases. When the pressure reaches 1 MPa, the F_MAX_ of the Neurotentacle approaches that of the control tungsten wire, indicating that the Neurotentacle possesses sufficient buckling resistance to enable reliable insertion into brain tissue.

The “F_L_‐Z” test was conducted for 10 cycles from 0.1 to 2 MPa, and the F_MAX_ at different pressures was recorded. As shown in Figure 3D, there is a clear positive correlation between F_MAX_ and pressure. It indicates that the strength of the Neurotentacle improves notably with internal pressure. At a pressure of 1 MPa, the F_MAX_ has risen to 1.2 mN, more than an order of magnitude above the unfilled state (80 µN). The same tests were performed with more Neurotentacles and the results were highly consistent (Figure S6, Supporting Information). The average result for all the tested probes is in agreement with that of a single probe, indicating a good mechanical consistency among different Neurotentacles.

To evaluate the bending behavior of the Neurotentacle under identical loading conditions at different pressure levels, radial and axial forces were applied separately at the tip of the Neurotentacle in two independent experiments (Figure S7 and Movie S2, Supporting Information). The results are shown in Figure 3E. Under the same radial force, the average axial deviation angle decreased from 22.2° at 0.1 MPa to 13.2° at 1.4 MPa. Similarly, under the same axial force, the average axial deviation angle decreased from 17.9° to 1.4° over the same pressure range. These results demonstrate that hydraulic actuation markedly improves the stiffness of Neurotentacles, providing sufficient rigidity for precise implantation.

### 2.5 High Robustness of Neurotentacles

To address concerns about the potential for rupture during implantation, fatigue tests were performed to verify the robustness of the Neurotentacles under high internal pressures. The displacement required for the probe to reach F_MAX_ at a given pressure was first determined. Then, the probe was rapidly dropped from the origin (0 µm) to the position of F_MAX_, and the loaded force at that point was recorded. The experiments were repeated 100 times at 1 and 2 MPa, respectively. As shown in Figure 3F, the same Neurotentacle exhibits a negligible decrease in loaded force after a total of 200 tests, demonstrating that the microchannel was still intact. The high robustness of the Neurotentacles ensures their safety during insertion and allows them to be implanted repeatedly.

### 2.6 Critical Pressure and Force for Inserting Neurotentacles into the Brain‐Like Gel

To test whether the enhanced Neurotentacle can penetrate the brain tissue and how much the critical pressure is for insertion, we performed simulated tests in the 0.6% agar gel. The experimental setup is shown in Figure S8 (Supporting Information). The test pressure range is from 0.1 MPa to 1 MPa. The results (Figure 3G; Movie S3, Supporting Information) showed that the Neurotentacle was unable to penetrate the gel at pressures of 0.1, 0.2, and 0.4 MPa. At pressures of 0.6 and 0.8 MPa, the Neurotentacle could penetrate the gel with some probability, which may be due to the inhomogeneous surface morphology of the agar gel. When the pressure reaches 1 MPa, the Neurotentacle can be inserted into the gel successfully in every test. Thus, the critical pressure for penetrating the brain‐like gel should be under 1 MPa, much less than the pressure resistance (≈2 MPa) of the Neurotentacles. This suggests that the Neurotentacles are strong enough to cope with more complex situations in the brain tissue.

To further investigate the critical force for the 1‐MPa Neurotentacles to penetrate brain tissue, the relationship between the loaded force and downward displacement was measured on the gel, as shown in Figure S9 (Supporting Information). The results of multiple tests were consistent (Figure 3H). The loaded force on the probes first rose and then suddenly fell with displacement. Unlike in Figure 3B, the loaded force rises more slowly, probably due to the deformation of the soft gel partially compensating for the increase in displacement. At ≈0.35 mN, the loaded force dropped abruptly, which indicated that the probes had penetrated the surface of the gel. This further demonstrates that the Neurotentacles are strong enough to penetrate the brain‐like gel, as it has an MLF of 1.2 mN at 1 MPa. In addition, we found that after breaking through the surface, the force required for traveling inside the gel is extremely small (Figure 3I). It is only ≈50µN, even lower than the MLF of unfilled Neurotentacles.

### 2.7 Slight Volume Increase of the Neurotentacle under Pressure

**Figure 4:**
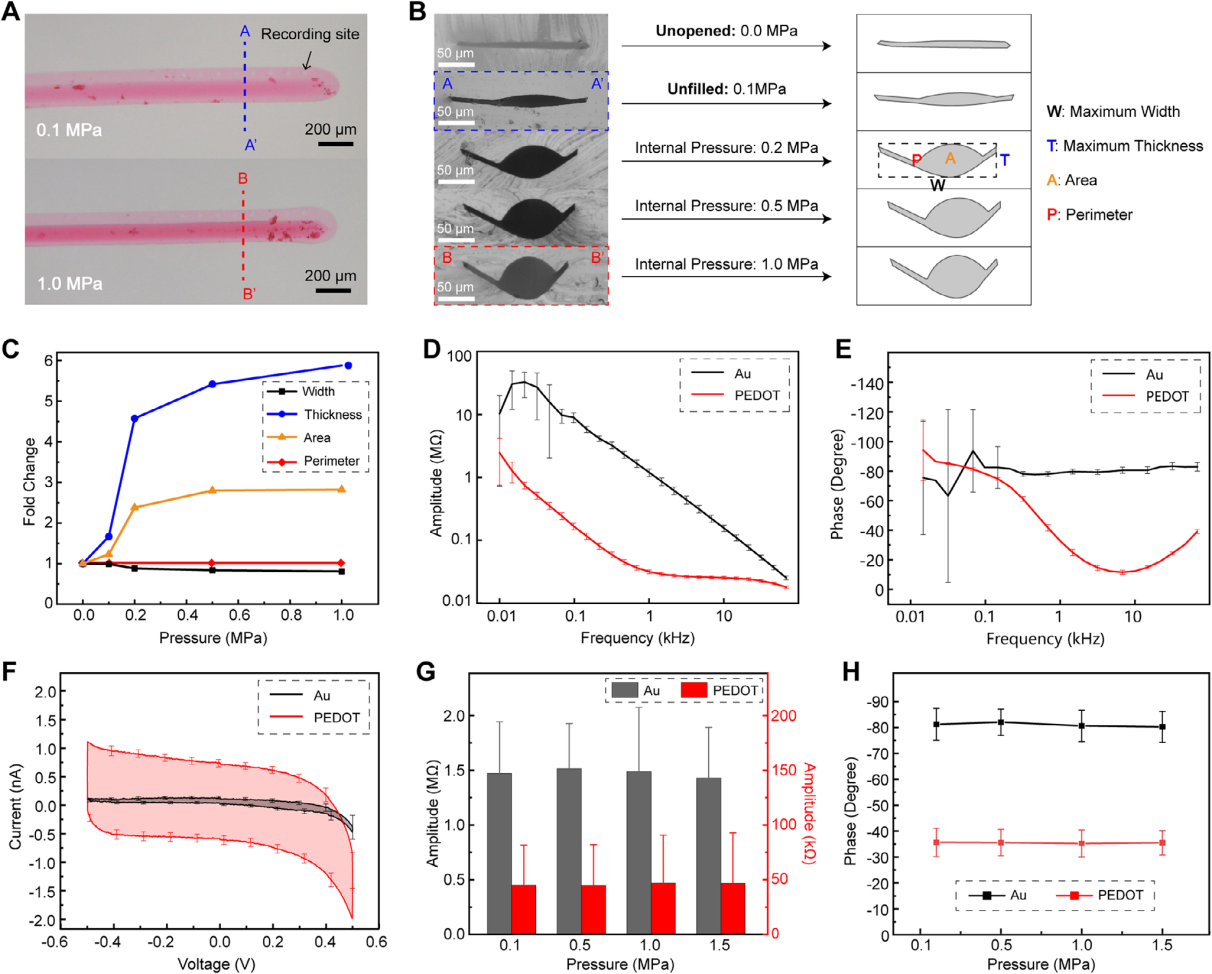
Morphology and electrochemical characterization of Neurotentacles. A) Obtained models of Neurotentacles under different pressures based on the flip‐mould of PDMS. B) Cross‐sections of Neurotentacles at different pressures. C) The relationship between the cross‐sectional parameters of Neurotentacles and the pressure. D–H) Electrochemical characterization of the recording sites on Neurotentacles before and after modification with PEDOT. (D) Frequency‐impedance spectra (n = 20). E) Frequency‐phase spectra (n = 20). F) Cyclic voltammetry (CV) tests (n = 20). G) Effect of varied pressure on impedance at 1 kHz (n = 14). H) Effect of varied pressure on phase at 1 kHz (n = 14). Data in (D–H) are means ± SD.

The Neurotentacle can be implanted without the use of rigid tools or temporary materials, thereby preventing a notable increase in volume during the implantation process. Upon application of pressure, the Neurotentacle will change its morphology due to the expansion of the microchannel, accompanied by a slight increase in volume. The cross‐sections of the Neurotentacle in different states were obtained using a flip mold of polydimethylsiloxane (PDMS) (**Figure** **4**A; Figure S10, Supporting Information). There are five different pressure states: 0.0 MPa (the microchannel is unopened), 0.1 MPa (the microchannel is open but not filled), 0.2, 0.5, and 1 MPa.

The extracted cross‐sections under different states are shown in Figure 4B. At a pressure of 0.1 MPa, the area of the cross‐section increases slightly compared to 0 MPa, but the overall shape remains as a sheet. At 0.2 MPa, there is a notable change in the cross‐sectional morphology and the microchannel turns into an elliptical shape. At 0.5 and 1 MPa, the microchannel is getting closer to circular and the area increases slightly. We extracted the parameters of each cross‐section by software and defined its maximum width (W), maximum thickness (T), Perimeter (P), and area (A), as shown in Table S1 (Supporting Information). Since Young's modulus of PI is as high as GPa,^[^ ^13^ ^]^ the Neurotentacle is unlikely to undergo plastic deformation under MPa‐level pressures and can reliably return to its original state after the process of liquid injection and withdrawal, as shown in Figure S11 and Movie S4 (Supporting Information). Thus, the perimeter of the probe should be relatively constant. This was used to calibrate the other dimensions of the cross‐sections. The results are shown in Table S2 (Supporting Information).

Defining the value at 0 MPa as 1, the trend of each cross‐section parameter with pressure is shown in Figure 4C. As the pressure rises, the width decreases, the thickness increases, and ultimately the cross‐sectional area is enlarged. At a pressure of 1 MPa, although the electrode thickness increases almost five times, the final cross‐sectional area only rises by a factor of ≈1.8 due to the reduced width. A typical shuttle based on the IFAT method has a characteristic dimension of ≈100 µm,^[^ ^2^, ^3^, ^22^, ^23^, ^24^, ^25^, ^35^, ^36^, ^37^, ^38^ ^]^ as shown in Table S3 (Supporting Information). The cross‐sectional areas of 100 and 50 µm tungsten needles are 7854 and 1963 µm^2^, respectively. When assembled with the flexible probe, their theoretical minimum areas become 9258 and 3367 µm^2^ by adding the probe's cross‐section (1404 µm^2^ at 0 MPa, Table S1, Supporting Information). This implies that the insertion volume of the Neurotentacle is reduced by ≈60% compared to the 100 µm needle‐assisted probe and is slightly larger than that of the 50 µm needle‐assisted probe. Furthermore, the implantation damage might be even lower if inserting the Neurotentacle with varied pressure, according to Figure 3I.

### 2.8 Stable Electrochemical Properties of Neurotentacle under Varied Pressures

Impedance spectroscopy and cyclic voltammetry (CV) are commonly used to characterize the electrochemical properties of neural electrodes. For electrodes of the same size, lower impedance is beneficial for recording high‐quality signals. Interface modification is a widely adopted approach to effectively lower electrode impedance. In this study, Poly(3,4‐ethylenedioxythiophene) (PEDOT) was applied to the recording sites of the Neurotentacles to improve their interface properties.

The frequency‐impedance spectra in Figure 4D illustrate that the average impedance amplitude of the electrodes decreased substantially after being modified with PEDOT. At 1 kHz, the amplitude dropped from 1.36 ± 0.35 MΩ to 33.5 ± 10.8 kΩ. The frequency‐phase spectra (Figure 4E) and CV curves (Figure 4F) can further validate the effectiveness of PEDOT modification from another perspective. There is no notable difference between the electrochemical performance of the Neurotentacles and that of normal PI probes.^[^ ^6^ ^]^

Given that the Neurotentacle probe undergoes structural changes following liquid injection into its internal microchannels, such deformations could potentially impact or compromise the functionality of the electrodes. To investigate this, we assessed the electrode impedance under varying internal pressures. As shown in Figure 4G,H, the results indicate that electrode impedance remains stable across a range of pressure levels, both before and after modification. This suggests that the electrochemical properties of the Neurotentacles are sufficiently resilient and stable to withstand volume changes within the microchannels induced by pressure variations.

### 2.9 Stable and High‐Quality in Vivo Recording with Neurotentacles

**Figure 5:**
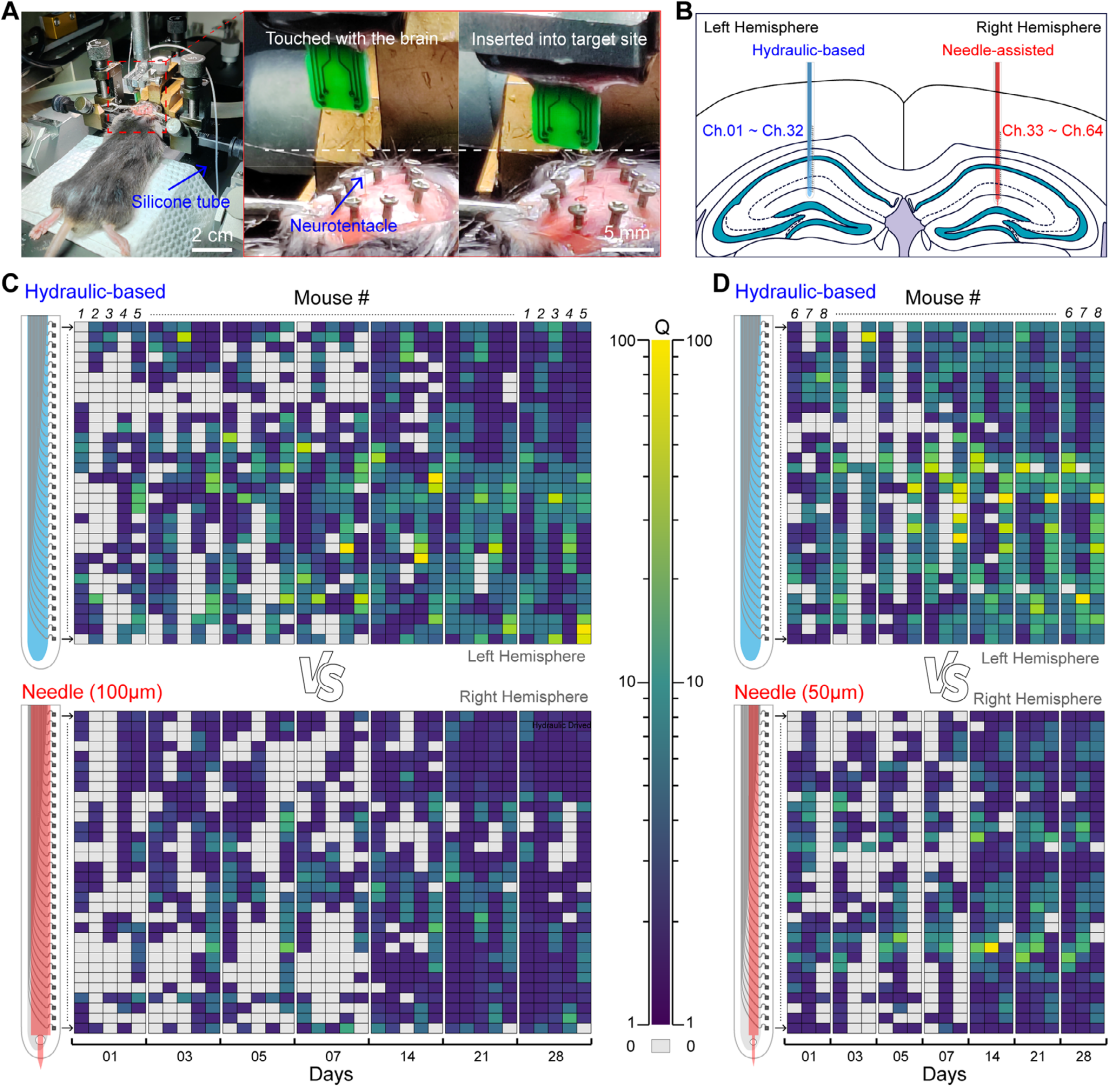
In vivo recordings of hydraulic‐based and needle‐assisted Neurotentacles in mice. A) Implantation of a Neurotentacle in the left hemisphere of the mouse. B) Schematic illustration of the implantation sites for hydraulic‐based and needle‐assisted Neurotentacles. C) The Q values of 320 channels over 7 sessions across 28 days in 5 mice (mouse #1 – #5), with hydraulic‐based implantation (top) and 100 µm needle‐assisted implantation (bottom). D) The Q values of 192 channels over 7 sessions across 28 days in 3 mice (mouse #6 – #8), with hydraulic‐based implantation (top) and 50 µm needle‐assisted implantation (bottom).

To evaluate the stability of the neurotentacles to obtain the electrophysiological signals of neurons, the hippocampus CA1 region, which is the most classic area in the field of electrophysiological recording, was selected as the brain area for probe implantation in this stage. To increase the amount of recorded data, the number of electrode channels on the Neurotentacle was extended to 32, with the spacing reduced to 50 µm (Figure S12, Supporting Information). The Neurotentacle probe was implanted into the left hemisphere of the mouse. As shown in **Figure** **5**A, the Neurotentacle was carried on a stereotaxic device for the implantation as conveniently as a rigid probe. Saline was used as the internal liquid. At a pressure of 1 MPa, the Neurotentacle could be easily inserted into the brain tissue. The insertion speed was 100 µm s^−1^ and the insertion depth was ≈3 mm. Then the saline in the Neurotentacle was pumped out to recover its flexibility. In the right hemisphere, the Neurotentacle probe was no longer implanted using hydraulic pressure but instead was inserted with tungsten needles of 100 or 50 µm in diameter, providing a more rigorous control (Figure 5B). Holes with diameters ranging from 30 to 40 µm were created at the front end of the Neurotentacle to accommodate the needles (Figure S13, Supporting Information). Additionally, a specialized implantation tool was designed using 3D printing technology to ensure consistency and reliability (Figure S14, Supporting Information). The same insertion speed and depth were used with the needle‐assisted implantation method.

Electrophysiological signal recordings were conducted on postoperative days 1, 3, 5, 7, 14, 21, and 28 (Figure S15, Supporting Information). Four indicators were used to assess the quality of the recorded signals: the number of functional channels (channels capable of isolating neurons), the number of isolatable neurons (sorted units or neuron yield), the signal‐to‐noise ratio (SNR), and a quality factor (Q) defined in this study. The quality factor (Q) is calculated as the product of the average SNR and the total sorted units across all functional channels, divided by the total number of channels. Therefore, Q serves as a comprehensive factor that measures both the SNR and neuron yield. For a single channel, Q is the product of its SNR and neuron yield.

The Q values of recorded signals from 320 channels across 5 mice in the 100 µm needle‐assisted control group, and from 192 channels across 3 mice in the 50 µm needle‐assisted implantation control group, over 28 days are shown in Figure 5C,D, respectively. These results clearly demonstrate that, over a 4‐week period, the hydraulic‐based probes exhibit a notable advantage in signal quality, regardless of whether the control group employs 100 or 50 µm needles for implantation. Additionally, it is evident that, after day 14, the signal quality in all experimental and control groups showed a noticeable improvement compared to previous recordings.

**Figure 6:**
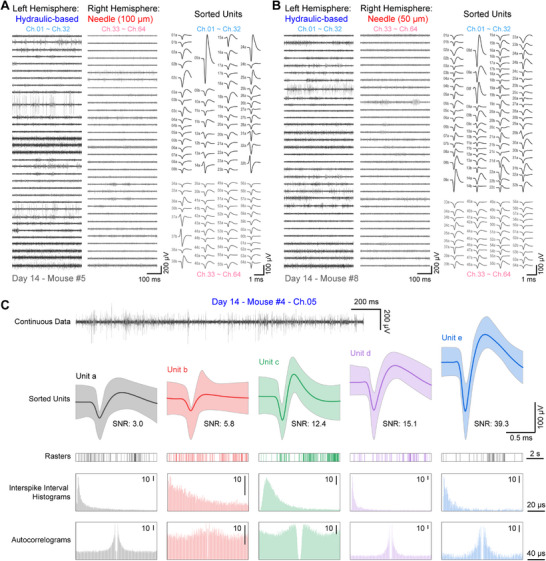
Some example data from mice on day 14. A) Continuous data and sorted units from mouse #5 on day 14. The hydraulic‐based probe was in the left hemisphere, corresponding to channels 01–32, and the 100 µm needle‐assisted probe was in the right hemisphere, corresponding to channels 33–64. B) Continuous data and sorted units from mouse #8 on day 14. The hydraulic‐based probe was in the left hemisphere, corresponding to channels 01–32, and the 50 µm needle‐assisted probe was in the right hemisphere, corresponding to channels 33–64. C) An example channel with excellent recording quality from mouse #4 on day 14. From top to bottom, the figure shows the filtered continuous data, sorted neuron units, and the corresponding rasters, interspike interval histograms, and autocorrelograms for each sorted unit.

The results in **Figure** **6** present some examples of data recorded from mice on day 14. Figure 6A shows the continuous data of recorded signals and the sorted units from hydraulic‐based and 100 µm needle‐assisted probes in the left and right hemispheres. The hydraulic‐based probe yielded 30 functional channels, with 51 sorted units and an average SNR of 7.84. In contrast, the 100 µm needle‐assisted probe produced 28 functional channels and 42 units, but with a lower average SNR of 3.53. The Q values for the two probes were 12.5 and 4.63, with a difference of ≈3 fold. Figure 6B presents the corresponding comparison between hydraulic‐based and 50 µm needle‐assisted probes. The Q values for the two probes were 15.2 and 3.66, with a difference of over 4 fold. These results further highlight that hydraulic‐based Neurotentacles can record a substantially higher number of neurons with better SNR. Additionally, we have observed multiple instances where the hydraulic‐based Neurotentacle has been able to sort more than five units from a single channel, with one such example shown in Figure 6C. This also demonstrates the excellent recording quality of the hydraulic‐based Neurotentacle.

**Figure 7:**
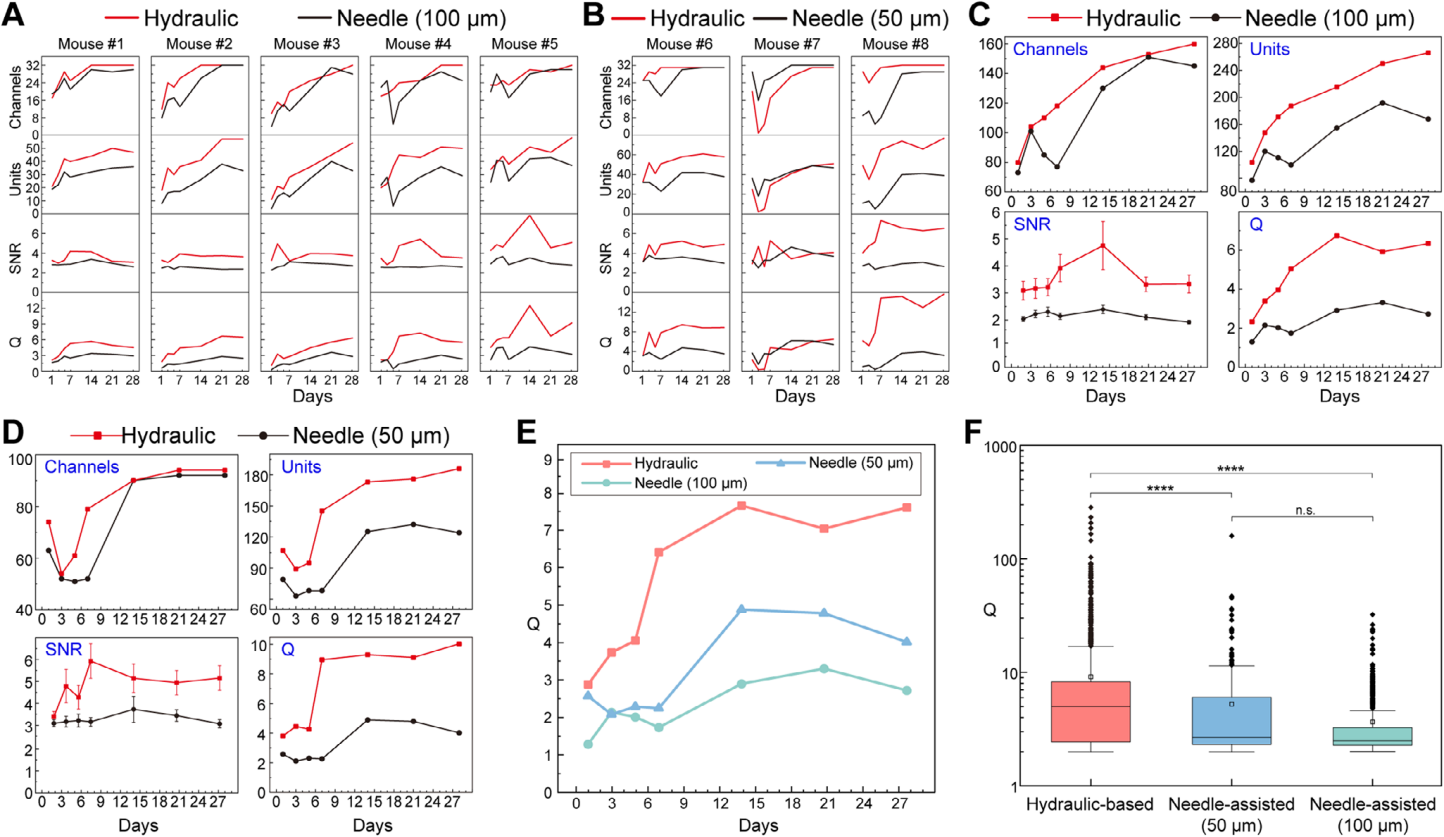
Comparison of signal quality between hydraulic‐based and needle‐assisted probes. A) Comparison between hydraulic‐based probe and 100 µm needle‐assisted probe (mouse #1‐ mouse #5). B) Comparison between hydraulic‐based probe and 50 µm needle‐assisted probe (mouse #6‐ mouse #8). C) Comparison between hydraulic‐based probe and 100 µm needle‐assisted probe (5 mice). D) Comparison between hydraulic‐based probe and 100 µm needle‐assisted probe (3 mice). E) Changes in the Q values of hydraulic‐based and needle‐assisted probes over time. F) Statistical analysis of differences between hydraulic‐based (n = 1414), 100 µm needle‐assisted (n = 762), and 50 µm needle‐assisted probes(n = 492). The data are expressed as the means ± SEMs. ^****^ *P* < 0.0001; n.s., not significant.

Furthermore, we present in **Figure** **7** the time‐dependent changes in signal quality metrics for all experimental mice over the 28 day period, including the number of functional channels, sorted units, average SNR, and Q value. Figure 7A presents a comparison of the results from the hydraulic‐based probe and the 100 µm needle‐assisted probe across five mice. It is evident that all metrics for the hydraulic‐based probe substantially outperform those of the needle‐assisted probe. Similarly, in Figure 7B, the hydraulic‐based probe also demonstrates superior performance when compared to the 50 µm needle‐assisted probe. Except for mouse #7, where the hydraulic‐based probe performed relatively poorly in the earlier recordings, it showed a remarkable improvement and ultimately surpassed the 50 µm needle‐assisted probe by day 28.

The results from the five mice in Figure 7A are summarized in Figure 7C. Overall, the hydraulic‐based probe maintains a substantial advantage over the 100 µm needle‐assisted probe. The signal quality of the hydraulic‐based probe steadily increased over the first 14 days, reaching a high level, and then remained stable. Compared to Day 1, the overall Q value of the hydraulic‐based probe increased from 2.34 to 6.73 on Day 14, nearly threefold its initial value. In contrast, the signal quality of the 100 µm needle‐assisted probe experienced a decline between days 5 and 7. It then peaked on day 14 but showed a slight decrease thereafter. For the overall comparison shown in Figure 7D between the hydraulic‐based probe and the 50 µm needle‐assisted probe, the hydraulic‐based probe similarly exhibited superior signal quality. Its signal quality reached a high level by day 7 and remained stable thereafter. In contrast, the signal quality of the 50 µm needle‐assisted probe experienced a decline between days 3 and 7. It peaked on day 14, followed by a slight downward trend.

The changes in the Q values over time for all hydraulic‐based (8 mice), 100 µm needle‐assisted (5 mice), and 50 µm needle‐assisted (3 mice) probes are presented in Figure 7E. It can be clearly observed that the hydraulic‐based probe consistently shows better signal quality compared to the needle‐assisted probes. Finally, in Figure 7F, the results from all recordings over 28 days are summarized, and categorized into hydraulic‐based, 100 µm needle‐assisted, and 50 µm needle‐assisted probes. Statistical analysis has confirmed a significant difference in signal quality between the hydraulic‐based and needle‐assisted probes.

### 2.10 Ultra‐Low Invasiveness of Neurotentacles

To evaluate the acute implantation damage of the Neurotentacles, another series of control experiments were conducted, comparing the hydraulic‐based probe with the 100 and 50 µm needle‐assisted probes. Four mice were used in each group. The surgical conditions were consistent with those used in the previous in vivo recording experiments.

**Figure 8:**
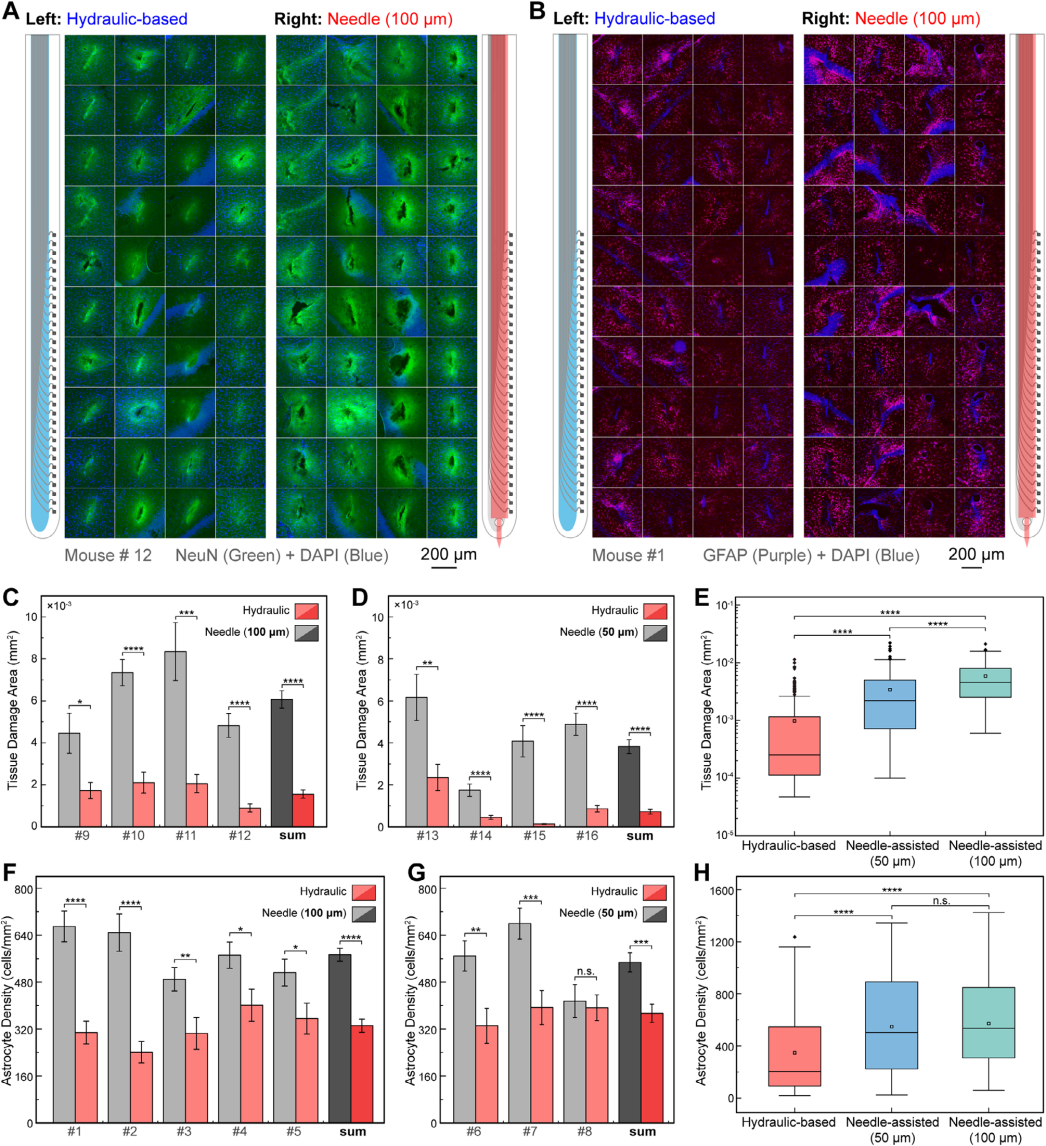
Acute tissue damage and chronic immune response of the Neurotentacle. A) Acute brain sections (horizontal) from mouse #12 showing hydraulic‐based probe (left) and 100 µm needle‐assisted probe (right) (green for NeuN, blue for DAPI). B) Chronic brain sections (horizontal) from mouse #1 after one month, showing hydraulic‐based probe (left) and 100 µm needle‐assisted probe (right) (purple for GFAP, blue for DAPI). C) Quantitative assessment of acute damage in four mice (mouse #9‐#12, n = 17, 31, 18, 43, respectively) with hydraulic‐based probes and 100 µm needle‐assisted probes. D) Quantitative assessment of acute damage in four mice (mouse #13‐#16, n = 21, 52, 46, 44, respectively) with hydraulic‐based probes and 50 µm needle‐assisted probes. E) Overall comparison of acute damage between hydraulic‐based probe (n = 267), 50 µm needle‐assisted probe (n = 158), and 100 µm needle‐assisted probe (n = 109). F) Quantitative assessment of chronic immune response in five mice (mouse #1‐#5, n = 42, 25, 39, 46, respectively) with hydraulic‐based probes and 100 µm needle‐assisted probes. G) Quantitative assessment of chronic immune response in three mice (mouse #6‐#8, n = 37, 38, 44, respectively) with hydraulic‐based probes and 50 µm needle‐assisted probes. H) Overall comparison of chronic immune response between hydraulic‐based probe (n = 314), 50 µm needle‐assisted probe (n = 119), and 100 µm needle‐assisted probe (n = 195). Data in (C‐H) are means ± SEM. ^*^ *P* < 0.05; ^**^ *P* < 0.01; ^***^ *P* < 0.001; ^****^ *P* < 0.0001; n.s., not significant.

After the implantation surgery was completed, the mice were immediately subjected to histological analysis, including perfusion, sectioning, and staining. To avoid potential misrepresentation caused by angle variations in histological flat images, all horizontal brain slices within the implantation depth for each mouse were analyzed (excluding any damaged slices). Representative results from one mouse in the 100 µm control group are shown in **Figure** **8**A, with results from the 50 µm control group provided in Figure S16 (Supporting Information).

From Figure 8A, it is clear that the 100 µm needle‐assisted probes left prominent cavity regions in most brain slices, with only a few slices showing faint traces. In contrast, the hydraulic‐based probes primarily left only faint traces, with cavities observed in only a few brain sections. Similarly, in Figure S16 (Supporting Information), the hydraulic‐based probes left fewer cavity regions compared to the 50 µm needle‐assisted probes. These results clearly indicate that hydraulic‐based probes cause less acute damage compared to needle‐assisted probes (whether 50 or 100 µm). Furthermore, the acute damage was more accurately quantified by measuring the area of the cavity regions or the maximum width of the shallow traces on the brain slices (Figure S17, Supporting Information). The results in Figure 8C,D show that, both individually and collectively, the damage caused by the hydraulic‐based probe is noticeably smaller than that caused by either the 100 or 50 µm needle‐assisted probes, with statistically significant differences observed. Overall, the average damage caused by the 100 µm needle‐assisted probe was 5834.5 µm^2^, while the hydraulic‐based probe caused an average damage of ≈1500.8 µm^2^, reducing the damage by ≈74.4%. For the 50 µm needle‐assisted and hydraulic‐based probes, the average damage was 3666.2 and 690.1 µm^2^, respectively, showing an 81.1% reduction with the hydraulic probe. In Figure 8E, all acute brain slices are classified into three categories according to the type of probe: hydraulic‐based (8 mice), 100 µm needle‐assisted (4 mice), and 50 µm needle‐assisted probes (4 mice). It can be seen that not only is there a significant difference between the hydraulic‐based and needle‐assisted probes, but also between the 50 and 100 µm needle‐assisted probes.

To further evaluate the chronic immune response of the Neurotentacles on tissue, the mice used for in vivo recordings were subjected to histological analysis one month after probe implantation. Representative results from one mouse in the 100 µm control group are presented in Figure 8B, with corresponding results from the 50 µm control group shown in Figure S18 (Supporting Information). Compared to the needle‐assisted control group, the number of astrocytes (purple) in the hydraulic‐based probe group was notably lower. Furthermore, the number of astrocytes in a 500 × 500 µm^2^ square region surrounding the probe implantation site on each brain slice was counted for quantitative analysis (Figure S19, Supporting Information). The results in Figure 8F,G show that, except for one mouse in the 50 µm control group, the chronic immune response induced by the hydraulic‐based probe is significantly lower than that caused by the needle‐assisted probes, both 50 and 100 µm, in other individuals and overall. Compared to the 100 and 50 µm needle‐assisted probes, the hydraulic probe reduced astrocyte numbers by ≈42.1% and 31.8%, respectively. Finally, the chronic histological results were categorized into three groups—hydraulic‐based (8 mice), 100 µm needle‐assisted (5 mice), and 50 µm needle‐assisted probes (3 mice) (Figure 8H). It was observed that the chronic immune response induced by the hydraulic‐based probe was significantly lower than that caused by the needle‐assisted probes. However, no significant difference was found between the 50 µm and 100 µm needle‐assisted probes.

## 3 Discussion

### 3.1 Advantages of Using the FMAX to Assess the Strength of Neurotentacles

The Euler buckling load (F_B_) is often used to characterize the buckling strength of neural probes. It can be denoted as:^[^ ^39^, ^40^ ^]^

(1) $$F_{B}=\frac{\pi^{2}EI}{{\left(k_{e}L\right)}^{2}}$$

where *E* is Young's modulus, *L* is the effective length, *k_e_* refers to the effective length coefficient that depends on the boundary conditions and is on the order of 1, and *I* is the moment of inertia and it is related to the shape and size of the section. However, in this paper, we use F_MAX_ rather than F_B_ to describe the variable strength of the Neurotentacle. First, although F_MAX_ does not directly equal the buckling force (F_B_), it represents the peak value in the F_L_‐Z curve and thus correlates linearly with F_B_. Second, when evaluating F_B_ or F_MAX_, the primary goal is to determine whether the probe will buckle under the insertion force (F_IN_). As shown in Figure S20 (Supporting Information), when the F_L_‐Z curve follows a typical buckling profile, using F_B_ as the evaluation metric is appropriate. However, for our stiffness‐tunable probe, the measured F_L_‐Z curve typically exhibits a peak (F_MAX_) followed by a gradual decrease, as shown in Figure 2A, suggesting that F_B_ is lower than F_MAX_. Under such conditions, as shown in Figure S20 (Supporting Information), whether the probe can penetrate without buckling is determined by the relationship between F_MAX_ and F_IN_, rather than F_B_ and F_IN_.

Moreover, from a practical perspective, measuring F_B_ typically involves inducing buckling in the probe, which may result in permanent deformation or structural damage. In contrast, the measurement of F_MAX_ is a gentler process that avoids irreversible damage. This allows the same Neurotentacle to be tested repeatedly under different conditions, with no mutual influence among the results and no risk of breaking the electrode traces. Considering both theoretical and practical aspects, we believe that F_MAX_ serves as a more appropriate indicator in our study for assessing whether the probe will bend during insertion.

### 3.2 Advantages of Adhesion‐Based Ultra‐Thin Microchannel Fabrication

The traditional sacrificial layer method is commonly used in microfluidic device fabrication due to its ability to precisely control both the width and height of microchannels. This technique involves forming the desired microchannel with a sacrificial material that is later dissolved. While effective, it introduces challenges when applied to implantable probes, particularly in managing high temperatures required for polyimide (PI) fabrication. The need for a heat‐resistant, removable sacrificial material complicates the process. Moreover, the sacrificial method tends to result in microchannels with predefined dimensions, which may increase the overall volume of the probe and potentially contribute to chronic immune responses. In contrast, our adhesion‐based microchannel fabrication technique simplifies the process while providing the strength required for implantation. While it doesn't provide the same precision in controlling the channel dimensions, this approach reduces the overall probe volume, minimizing chronic immune responses.

### 3.3 Factors Affecting the Maximum Strength of Neurotentacles

As depicted in Figure 3D, there is a clear positive correlation between the strength of the Neurotentacle and the applied liquid pressure. However, the strength increase is gradually slowing down as the pressure rises. We speculate that this is because the pressure regulates the strength primarily by causing changes in the probe footprint and secondarily by altering Young's modulus. For the Neurotentacle, both Young's modulus and the footprint are variable with pressure. As shown in Figure 4B, the probe size increases rapidly when the pressure begins to rise, which may result in faster growth of strength at first in Figure 3B. After that the probe size tends to stabilize and the strength increases more slowly with the equivalent of Young's modulus. Although increasing the liquid pressure can theoretically keep improving the strength of the probe, actually exceeding a pressure of 3 MPa will challenge the sealing of the whole liquid‐connected chamber. What is more critical in determining the upper limit of the strength is the footprint of the probe, i.e., the size of the microchannel.

### 3.4 Minimized Probe Displacement through Controlled Deformation of the Neurotentacle

The Neurotentacle's ability to recover to a thin and soft state helps reduce its dimension and stiffness after implantation, thereby minimizing long‐term mechanical disturbance to the tissue and improving biocompatibility. Although this structural transformation may cause potential displacement during probe recovery, possibly affecting positioning accuracy, such impact is very limited for the following reasons:

Initially, as shown in Table S2 (Supporting Information), the probe exhibited slight variations in width and thickness, generally within 50 µm. Moreover, histological observations (Figure 8A) reveal no significant cavities along most of the implantation path, suggesting that the tissue deformation caused by insertion might be partially reversible as the probe recovers. Furthermore, the probe's design intentionally separates the microchannel region from the recording site region (Figure 1C). Recording sites are located outside the primary deformation zones, preserving their spatial stability during probe recovery. In combination, these factors help to minimize the relative displacement between the recording sites and surrounding tissue, ensuring stable and reliable neural signal acquisition in typical experimental conditions.

Nevertheless, for applications requiring exceptionally high spatial precision—such as targeting single neurons—even minor displacement warrants careful consideration. These cases may call for a more systematic characterization of deformation effects and optimization of probe geometry or implantation protocols to enhance positional stability.

### 3.5 Rationale for Selecting the Needle‐Assisted Approach Over the Coating‐Assisted Approach as the Control

In this study, the needle‐assisted implantation method was chosen as the control instead of the coating‐assisted approach due to its clear advantages and greater reliability. Unlike the coating‐assisted method, which requires rapid insertion to avoid premature coating dissolution, the needle‐assisted approach and Neurotentacle probe both allow precise control of insertion speed. Moreover, after implantation, the needle‐assisted method leaves only the flexible probe embedded in the tissue, while the coating‐assisted method inevitably results in residual materials that may introduce biochemical confounding factors affecting immune response and signal quality assessments. The needle‐assisted approach also benefits from standardized microneedle specifications, ensuring uniformity and reproducibility, whereas biodegradable coatings vary widely in composition and thickness without established standards. Additionally, temporary assemblies formed by the needle‐assisted method exhibit excellent mechanical stability and consistency, facilitating reliable implantation and scalable batch processing. In contrast, coating‐assisted assemblies are more sensitive to environmental factors, resulting in greater variability and potentially compromising experimental fairness. Taken together, these considerations highlight the suitability and advantages of the needle‐assisted approach as a control for assessing the Neurotentacle's performance in terms of tissue damage and recording quality.

### 3.6 Consistency between the Recording Quality, Acute Damage, and Chronic Immune Response of the Neurotentacles

Sections 2.9 and 2.10 present highly consistent results, demonstrating clear advantages of hydraulic‐based neural probes over needle‐assisted probes. These advantages include better recording quality, lower acute injury, and reduced chronic immune responses. Due to the rigorous control groups implemented in the study, with all conditions kept consistent except for the flexible probe implantation method, and a sufficiently large sample size, we can attribute these differences in results to the innovative minimally invasive implantation approach we proposed. Hydraulic‐driven neural tentacles, compared to traditional shuttle‐based implantation methods, result in lower acute implantation damage, leading to weaker immune responses during both the acute and chronic phases. As a consequence, superior signal quality is maintained throughout this period.

Although the comparison between the 100 µm needle‐assisted probes and the 50 µm needle‐assisted probes is not as strictly controlled as the comparison with the hydraulic‐based probes, some interesting observations can still be made from the overall comparison. For instance, the 100 µm needle‐assisted probes exhibited greater acute damage than the 50 µm needle‐assisted probes (Figure 8E), yet no significant difference was found between the two in terms of chronic immune response after one month (Figure 8H). In terms of signal recording quality, the 50 µm needle‐assisted probes appeared to have a slight advantage over the 100 µm needle‐assisted probes, but no significant difference was observed statistically (Figure 7F). It is speculated that this may be due to the relatively small difference in acute damage between the 100 and 50 µm needle‐assisted probes, which further emphasizes the importance of reducing damage during the implantation of flexible electrodes and highlights the significance of the work conducted.

While the present study focuses on the hippocampus, the proposed method holds potential for broader applications. Future work will explore its feasibility in more challenging regions, such as the brain‐stem—where surgical precision and biocompatibility are even more critical—to further demonstrate the significant advantages and broader application potential of the Neurotentacle compared to conventional flexible implantation methods.

### 3.7 Minimally Invasive Mechanism of the Neurotentacles

In the control groups set up in this study, identical flexible probes were used, but the insertion methods differed. However, the higher damage observed with the needle‐assisted approach does not seem to be solely attributed to differences in insertion volume. As mentioned in Section 2.7, the volume of the hydraulic‐based probe is reduced by over 60% compared to the 100 µm needle‐assisted probe. However, when compared to the 50 µm needle‐assisted probe, the volume of the hydraulic‐based probe is actually slightly larger.

We attribute the low invasiveness of the hydraulic‐based Neurotentacle to the following factors:

1. Probe shape: The hydraulic‐based Neurotentacle probe is a single, unified structure with a more uniform shape. In contrast, the microneedle‐assisted probe comprises a rigid needle and a flexible probe that is temporarily fixed together. The assembled tip exhibits a more complex geometry (Figure S13, Supporting Information), which can significantly influence tissue damage during insertion. Despite using finer needles, this configuration still has the potential to cause greater damage.
2. Material properties: The rigid needle, with a higher Young's modulus compared to the flexible Neurotentacle probe, is more likely to cause irreversible tissue damage during insertion, and the tissue may not fully recover after the needle is retracted. In contrast, the Neurotentacle probe, being flexible and surface‐compliant, may just displace tissue during insertion and allow it to rebound after the liquid is drained and the probe returns to its thin‐film state.
3. Retrieval process: After the flexible probe is implanted, the shuttle must be retracted, which can introduce secondary tissue damage. In contrast, our Neurotentacle probe does not require such a retrieval step.

### 3.8 Potential for Further Reduction in the Invasiveness of the Neurotentacles

Through in vitro and in vivo experiments, the Neurotentacle was demonstrated with low invasiveness. Actually, the invasiveness can be further reduced. We found that the F_MAX_ of Neurotentacle at 1 MPa is close to 1.2 mN (Figure 3B), but it needs only ≈0.43 mN to penetrate the brain‐like gel (Figure 3I). In addition, the applied pressure can be increased to more than 2 MPa. These results indicate that there is still room to reduce the footprint of the Neurotentacle, thus reducing the insertion damage. It requires a proper compromise between the size and the maximum strength of the probe.

Moreover, the force required for traveling inside the brain‐like gel is less than 100µN (Figure 3B). This suggests that it may be possible to penetrate the tissue with a higher pressure but move to the target depth with a lower pressure. We have validated it in the brain‐like gel, as shown in Figure S21 and Movie S5 (Supporting Information). The Neurotentacle successfully penetrated the gel at 1 MPa and continued insertion at 0.1 MPa, although a deviation from the straight trajectory was observed. This demonstrates the potential for extremely low‐invasive implantation using Neurotentacles. The trajectory deviation was caused by the complete release of internal pressure, which reduced the probe's stiffness to its lowest state and made it more prone to bending under frictional forces during insertion. This issue can be addressed by partially maintaining internal pressure or increasing the probe's thickness to enhance its buckling resistance, as well as by optimizing the probe tip geometry to reduce insertion resistance.

We also conducted a preliminary in vivo test where the Neurotentacle probe was inserted into brain tissue under 1 MPa pressure, with the liquid completely drained before insertion continued. However, this approach led to probe bending, likely due to the complexity of the brain environment, including the presence of blood vessels and other relatively rigid structures. In future studies, we will explore the optimal pressure required after tissue penetration to enable smooth continuation of probe insertion.

On the other hand, while all experimental and control groups in this study were implanted at a consistent speed (100 µm s^−1^), previous studies have shown that implantation speed can significantly affect the degree of invasiveness and tissue response.^[^ ^41^, ^42^, ^43^ ^]^ For example, Bjornsson et al. found that slow insertion (125 µm s^−1^) resulted in greater tissue strain and damage compared to fast insertion (2000 µm s^−1^). In contrast, Welkenhuysen et al. reported no significant differences in glial response, inflammation, or neuronal loss between insertion speeds of 10 and 100 µm s^−1^. Furthermore, Fiáth et al. showed that an ultra‐slow insertion speed (2 µm s^−1^) could achieve better acute recording quality and reduced tissue damage. In future work, we plan to systematically evaluate the effects of different insertion speeds on both tissue damage and signal quality. By jointly optimizing implantation speed, internal pressure, and probe geometry, we aim to investigate the potential for minimizing the invasiveness of the implantation process.

### 3.9 Potential of Probe Restiffening for Improved Maneuverability

One major limitation of traditional shuttle‐based probes is the lack of maneuverability after the shuttle is extracted, which prevents the probe from targeting different cell populations within the same neural structure. In contrast, the ability to re‐stiffen the Neurotentacle probe using hydraulic actuation offers a notable advantage, enabling more precise control over probe positioning. This feature not only addresses the maneuverability issue but also opens the possibility of sampling multiple cell populations in a single experiment, enhancing the scope of neural recording.

Although demonstrating this feature in a chronic experiment would offer valuable insights, such studies were outside the scope of the current research due to the technical complexity and extended timelines required. Nevertheless, the potential of probe restiffening for improved maneuverability remains an exciting direction for future research, which could substantially expand the capabilities and practical applications of neural recording technologies.

### 3.10 Finite Element Modeling of the Neurotentacle for Morphological and Mechanical Performance Analysis

To further investigate the mechanical behavior of the Neurotentacle and facilitate the optimization of its design in future work, we constructed simplified 2D finite element models using COMSOL Multiphysics for simulation purposes. These models focus on the structural deformation and axial stiffness variations of the probe under different hydraulic pressures. As shown in Figure S22 (Supporting Information), the simulation results preliminarily reveal the volume expansion induced by liquid injection and its impact on the overall mechanical performance, indicating that hydraulic actuation can effectively tune the probe's stiffness and potentially enhance its buckling resistance during insertion, which is consistent with our experimental observations. Although the current model remains simplified and does not fully capture the complex mechanisms of fluid‐structure interaction and dynamic 3D implantation, it provides a valuable theoretical foundation for future structural optimization. We plan to further refine the model in subsequent work to comprehensively investigate the relationship between structural parameters and implantation performance.

## 4 Conclusion

In this work, we present Neurotentacle, a hydraulically actuated, stiffness‐tunable neural probe that achieves minimally invasive implantation without rigid insertion tools or dissolvable coatings. Its ultrathin, hydraulically actuated structure enables real‐time stiffness modulation, ensuring reliable penetration during implantation and mechanical compliance afterward. This reduces both acute injury and chronic immune response while maintaining superior recording performance over four weeks. Unlike conventional flexible probes, Neurotentacle offers controllable deployment and the potential for restiffening, laying the foundation for adaptive, long‐term neural interfaces. With its simple fabrication, small footprint, and robust in vivo functionality, Neurotentacle represents a promising platform for next‐generation brain‐computer interfaces and intelligent bioelectronic systems.

## 5 Experimental Section

### Dilution of PI2611

The dilution for PI was referred to the previous work of Liu et al.^[^ ^34^ ^]^ The procedures are as follows: i) Mix the PI2611 prepolymer with the NMP solution at a mass ratio of 4:1. ii) Use a magnetic stirrer to make the mixture well blended. iii) Remove the air bubbles from the mixture in a vacuum environment. iv) Obtain the diluted PI2611 prepolymer.

### Fabrication of Neurotentacle

The processes for manufacturing the Neurotentacles are shown in Figure 2A,B and are described as follows: i) Spin‐coating (2000 r s^−1^) diluted PI2611 on the silicon wafer and baking (300 °C) to form the bottom PI layer. ii) Patterning the positive photoresist (AZ4620) on the bottom PI to define the microchannel regions. iii) Treating the surface with reactive ion etching (RIE, 90 s) to create a strongly adhesive region around the microchannels. iv) Patterning the negative photoresist (AR4340) with the microchannels being uncovered. v) Treating the microchannels with hydrophobic reagents (1H,1H,2H,2H‐tridecafluorooctyl) to further reduce the adhesion. vi) Spin‐coating (5000 r s^−1^) diluted PI2611 and baking (300 °C) to form the middle PI. vii) Treating the middle PI with RIE (O_2_, 30 s) to increase its adhesion. viii) Patterning the photoresist (AR4340), depositing the composite metal layers (Cr/Au/Cr), and lifting off the photoresist to obtain the metal traces and electrodes. ix) Spin‐coating (4000 r s^−1^) undiluted PI2611 and baking (300 °C) to form the top PI. x) Patterning the photoresist (AZ4620), RIE etching the PI, and lifting off the photoresist to define pads, recording sites, and probe profiles. xi) Etching the upper Cr layer on the pads and recording sites and annealing the wafer on a hot plate (350 °C) for 30 min. xii) Soaking the wafer in deionized water and using a tweezer to release the Neurotentacles from the substrate.

For the RIE treatment, the conditions used were as follows: oxygen gas, gas flow rate of 20 sccm, RF power of 300 W, and pressure of 9 Pa. For the hydrophobic treatment, 100 µL of hydrophobic reagents was dropped into the bottom of a glass vacuum chamber, and the samples were then placed inside. After evacuation, the chamber was maintained at room temperature for 4 h.

### Encapsulation of Neurotentacle

The procedures for encapsulating the Neurotentacle with water and electrical connectors are shown in Figure S4 (Supporting Information), and they are described as follows: i) Opening the microchannel from the rear end with a syringe needle (outer diameter: 0.18 mm). ii) Insert the syringe needle into the microchannel until it closely fits the inner PI wall. iii) Sealing the joint with UV glue. iv) Connect the balloon syringe pump to the syringe needle and inject liquid to open the whole microchannel. v) Cutting off the syringe with only the stainless‐steel needle left. vi) Electrically connecting the Neurotentacle to the PCB with gold ball bonding.^[^ ^6^, ^25^ ^]^ vii) Attaching a silicone tube (inner diameter: 0.3 mm, outer diameter: 0.8 mm) to the needle and sealing the joint with UV glue. viii) Connecting a syringe needle (outer diameter: 0.18 mm) to the other end of the silicone tube and sealing the joint. ix) Soldering the 8‐channel connector (pin pitch: 1.27 mm) or 32‐channel connector (pin pitch: 0.64 mm) to the PCB. x) Sealing the whole PCB with UV glue to form a waterproof and insulating encapsulation.

### Mechanical Testing of Neurotentacle

During testing the “load force‐displacement‐pressure” relationship for Neurotentacles, a motorized stage was used to carry the probe up and down with a minimum step distance of 1 µm, a microbalance was used to measure the load force with a minimum readability of 1µN, and the hydrometer on the syringe pump was used to monitor the internal pressure with a minimum scale of 0.1 MPa. All the loaded force test (F_L_‐Z) shown in Figure 3 was conducted in a quasi‐static manner. Specifically, the probe was lowered incrementally at 1 s intervals, with each step performed at a relatively high speed of 100 mm s^−1^, and the force was recorded after each step. The step sizes were as follows: 1 µm per step from 0 to 10 µm, 10 µm per step from 10 to 100 µm, and 100 µm per step beyond 100 µm. The brain‐like gel penetration test was performed using a similar stepwise approach, with a step size of 100 µm and the same speed of 100 mm s^−1^. For the axial deviation angle measurement, radial and axial forces were applied separately in independent experiments. The testing environment is illustrated in Figure S7 (Supporting Information). The magnitude of the radial load was 30 µN, and the axial load was 0.3 mN. During testing, either the radial or axial force was kept constant, and the probe's deviation angle from the axial direction was recorded under various hydraulic pressures (0.1, 0.2, 0.4, 0.6, 0.8, 1.0, 1.2, and 1.4 MPa). The observation window length was 2.7 mm for radial loading and 2 mm for axial loading. The recorded image data were processed and analyzed using ImageJ software. The analysis workflow is illustrated in Figure S7 (Supporting Information). First, the images were converted to 8‐bit grayscale. Then, automatic thresholding was applied using the Yen mode. The angle between the main axis of the probe and the horizontal plane was subsequently measured. Finally, this angle was converted to the deviation angle relative to the probe's axial direction.

### Morphological Testing of Neurotentacle

The cross‐sections of the probe under different hydraulic pressures were acquired based on the flip‐mould of PDMS. The procedures are shown in Figure S10 (Supporting Information) and are described as follows: i) Placing a mold on the hot plate (20 °C) and holding the Neurotentacle vertically inside the mold. ii) Filling the mold with PDMS (Sylgard 184, Dow Corning, with the weight ratio of prepolymer to curing agent of 10:1) until the Neurotentacle was submerged ≈5 mm. iii) Curing PDMS at 120 °C for 10 min. iv) Removing the probe and taking the cured PDMS out of the mold. v) Cutting off the PDMS to expose the cross‐section at the depth of the recording sites. vi) Acquiring the cross‐section with an optical microscope. vii) Obtaining the cross‐sectional outline of the microchannel and calculating the dimensional parameters with software (Matlab).

### Electrochemical Characterization of Neurotentacle

An electrochemical workstation (CHI660D) was used to perform frequency‐impedance spectroscopy, cyclic voltammetry (CV), and modification, with the microelectrodes as the working electrodes and a Pt electrode as the counter electrode. The impedance and CV were measured in PBS solution. Poly(3,4‐ethylenedioxythiophene) (PEDOT) was deposited in an aqueous solution consisting of 0.02 M EDOT monomer and 0.1 M TsONa (sodium p‐toluene sulfonate) electrolyte using the constant current method with a current magnitude of 6.35 nA for 30 s per electrode.

### Animals

The surgical procedure was conducted on male mice of the C57BL/6J strain, which were 2 months old at the commencement of the experiments. Mice were housed in a specific pathogen‐free (SPF) barrier environment with a 12 h light/dark cycle control at 22 ± 1 °C and with food and water ad libitum. Animals were implanted with chronic flexible neural microelectrodes and single‐housed. Chronic electrophysiological recording experiments were conducted during the light phase. Prior to the commencement of the electrophysiological recording experiments, the animals were habituated to the experimenter by handling for a minimum of 3 consecutive days (10 min per day). Animal care and use were under the guidelines approved by the Animal Care and Use Committee in the Institute of Psychology, Chinese Academy of Sciences (SYXK20210018).

### Surgical Implantation of Flexible Neural Microelectrodes

Mice were initially anesthetized using isoflurane (induction: 5.0%, maintenance: 1.0%), before being secured within a stereotaxic frame (RWD Life Science, China) and eye ointment was subsequently applied. To evaluate the acute damage and the quality of in vivo recordings, a flexible probe introduced by the tungsten needle and a Neurotentacle were implanted in either the left or the right side of the CA1(coordinates: 1.82 mm posterior, 1.50 mm lateral, and 1.20–1.40 mm ventral from Bregma). Before acute insertion, the tips of the silicon probe were dipped in either DiI (saturated) ethanol solution and allowed to dry. The location of the probe tip, the area of damage, and the location of these microelectrodes can be determined based on DiI. Holes were drilled above the target brain regions. A tungsten‐needle‐guided flexible probe was implanted using the IFAT method with an insertion stable speed of 100 µm s^−1^ to avoid potential damage to local brain tissue. Then the tungsten needle was withdrawn at a speed of more than 100 µm s^−1^, leaving only the flexible probe in the brain (Figure S14, Supporting Information). The Neurotentacles were carried on a stereotaxic device for implantation (Figure 3D). The pressure inside the probe during insertion was 1 MPa, and the insertion speed was also 100 µm s^−1^. After reaching the specified depth, release the inside pressure to 0.1Mpa, and then cut the silicone tube at the headstage. The two implanted probes were fixed to the skull with miniature skull screws, cyanoacrylate glue, and blended dental cement. After implantation, antibiotics were applied.

### Chronic Electrophysiological Recordings and Data Analysis

Mice were allowed to recover for 4 days after surgery and then acclimated to the headstage and cables for several days before electrical recording began. To explore CA1 activity, subject mice were placed in the open‐field apparatus and allowed to acclimate for 10 min before starting in vivo recordings. Wideband electrophysiological data were recorded with a sampling rate of 40 kHz using the OmniPlex Neural Recording Data Acquisition System (Plexon Inc, USA). Subsequently, the raw wide‐band data were preprocessed using the Offline Sorter software (Plexon Inc, USA) and further analyzed through NeuroExplorer (Nex Technologies, USA). MATLAB (MathWorks, USA) was used to extract the processed data, and Origin (OriginLab Corporation, USA) was used for data visualization. To detect the spiking activity of single units, a standard fixed threshold method was employed, with the amplitude threshold set at 4 standard deviations. The units were then automatically separated by principal component analysis and cluster analysis.

The signal‐to‐noise ratio (SNR) tool was calculated by the Offline Sorter software using the following formula:

(2) $$SNR=\frac{\sigma_{\mathrm{signal}}^{2}}{\sigma_{\mathrm{noise}}^{2}}$$

where $\sigma_{\mathrm{signal}}^{2}$ is the variance of the signal and $\sigma_{\mathrm{noise}}^{2}$ is the variance of the noise. Since there were sorted units in the spike channels, $\sigma_{\mathrm{signal}}^{2}$ is defined as all sorted spikes, and $\sigma_{\mathrm{noise}}^{2}$ is defined as the data between all spikes. The SNR of a channel is determined by the highest SNR among its sorted units.

### Immunohistochemistry

Mice were anesthetized with isoflurane and perfused sequentially with phosphate‐buffered saline (PBS) followed by 4% paraformaldehyde (PFA). For post‐fixation, brains were incubated in PBS containing 30% sucrose until they sank to the bottom. Horizontal Cryostat sections (40‐µm perpendicular to the probes) were obtained using a cryostat (Leica CM3050S). 40 µm horizontal sections were collected from DV −0.20 to −2.0 mm for each mouse. Sections were washed in PBST 3 times (10 min each time) and then incubated with blocking solution (PBS containing 10% goat serum and 0.5% Triton X‐100) for 2 h at room temperature, and then treated with primary antibodies diluted with blocking solution overnight at 4 °C. The following primary antibodies were used: Rat anti‐NeuN (1:500; Oasis‐Biofarm, OB‐PRT013), and Rabbit anti‐GFAP (1:500; Oasis‐Biofarm, OB‐PRB005). After three washes in PBST (10 min each), sections were then incubated with species‐specific fluorophore‐conjugated secondary antibodies (1:1000, goat anti‐rat 488 nm, Oasis‐Biofarm, RT488 or 1:1000, goat anti‐rabbit Alexa Fluor 546, Invitrogen, A‐11010) in PBS for 2 h at room temperature. After washing in PBST 3 times (10 min each time), sections were stained with DAPI, washed with PBST, and transferred to microscope slides. Sections were imaged using an OLYMPUS VS200 fluorescence microscope (10 × objective lens).

### Quantitative Analysis of Acute Damage and Chronic Immune Response

The brain slice images were analyzed using ImageJ software. For acute slices, the area of the cavity or the maximum width of the shallow trace left by the probe insertion was measured. The image processing workflow is shown in Figure S17 (Supporting Information). The green channel was split from the original RGB image and converted to 8‐bit. The threshold was then adjusted (automatically), followed by measuring the area of the cavity or the width of the trace, depending on the type of damage. For chronic slices, the number of astrocytes within a 500 × 500 µm^2^ area surrounding the probe was calculated. The image processing workflow is shown in Figure S19 (Supporting Information). The red channel was split from the original RGB image and converted to 8‐bit. The threshold was then adjusted (using the intermodes method), followed by particle analysis.

### Quantification and Statistical Analysis

The experimental samples were processed by the experimenters in accordance with a double‐blind method. Statistical comparisons of the two groups of experimental data were performed using either a paired or an unpaired two‐tailed Student's *t*‐test. In cases where there were more than two groups of data, a one‐way or two‐way analysis of variance (ANOVA) was employed for statistical evaluation. In the case of ANOVA, the data were subjected to a Geisser‐Greenhouse correction to ensure equivariance of the differences observed and then subjected to a Bonferroni post hoc test for the purpose of making multiple comparisons between the groups in question. The sample size in our study was not predetermined by any statistical method but was similar to that used in previously published literature. All data presented in this study were expressed as mean ± standard error of the mean (SEM). The following significance levels were used: ^*^ *P* < 0.05, ^**^ *P* < 0.01, ^***^ *P* < 0.001, and ^****^ *P* < 0.0001. GraphPad Prism 10 (GraphPad Software, Inc.) and Origin 2018 were used for data visualization and statistical analyses. Detailed information on statistical analyses is provided in the figure legends.

## Conflict of Interest

The authors declare no conflict of interest.

## Supporting information



Supplemental Movie 1

Supplemental Movie 2

Supplemental Movie 3

Supplemental Movie 4

Supplemental Movie 5

Supporting Information

## Data Availability

The data that support the findings of this study are available from the corresponding author upon reasonable request.
