# Supplementary material for: Stiffness‐Tunable Neurotentacles for Minimally Invasive Implantation and Long‐Term Neural Activity Recordings
Source: Adv Sci (Weinh). 2025 Jul 21;12(36):e05100. doi: 10.1002/advs.202505100 (PMC12462962; doi:10.1002/advs.202505100)
Supplement: Supplementary file 6 — Supporting Information [file ADVS-12-e05100-s006.pdf]

ADVANCED SCIENCE
Open Access

Supporting Information

for *Adv. Sci.*, DOI 10.1002/advs.202505100

Stiffness-Tunable Neurotentacles for Minimally Invasive Implantation and Long-Term Neural Activity Recordings

Yang Wang, Xing Xu, Xiaowei Yang, Rongyu Tang, Ying Chen, Shan Zang, Yijun Wang, Jing Liang and Weihua Pei**

Supporting Information

Stiffness-tunable Neurotentacles for minimally invasive implantation and long-term neural activity recordings

*Yang Wang, Xing Xu, Xiaowei Yang, Rongyu Tang, Ying Chen, Shan Zang, Yijun Wang, Jing Liang**, and Weihua Pei***

*Corresponding author. Email: peiwh@semi.ac.cn, liangj@psych.ac.cn

Yang Wang and Xing Xu contributed equally to this work.

This supplementary material includes:

Figures S1 to S22
Tables S1 to S3
Movies S1 to S5

Figure S1. The relationship between spin-coating speed and film thickness for diluted PI2611(mean ± SD)

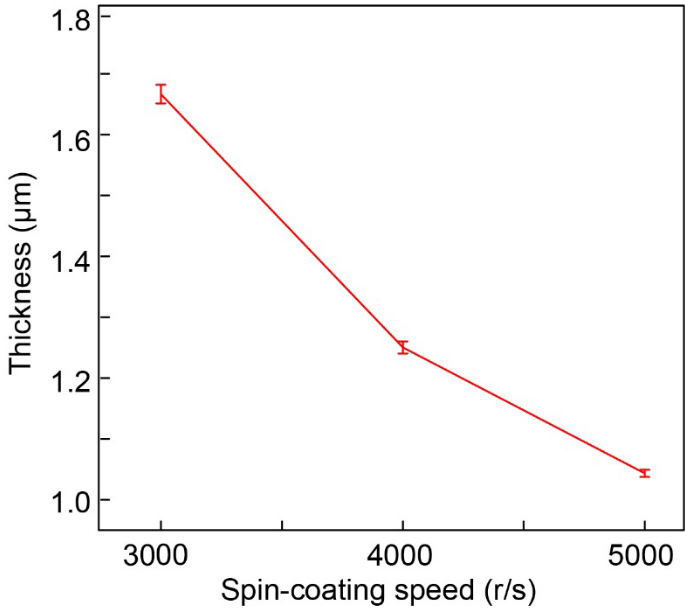

Figure S2. Self-bending of the neurotentacles due to uneven PI thickness distributions above and below the metal layer

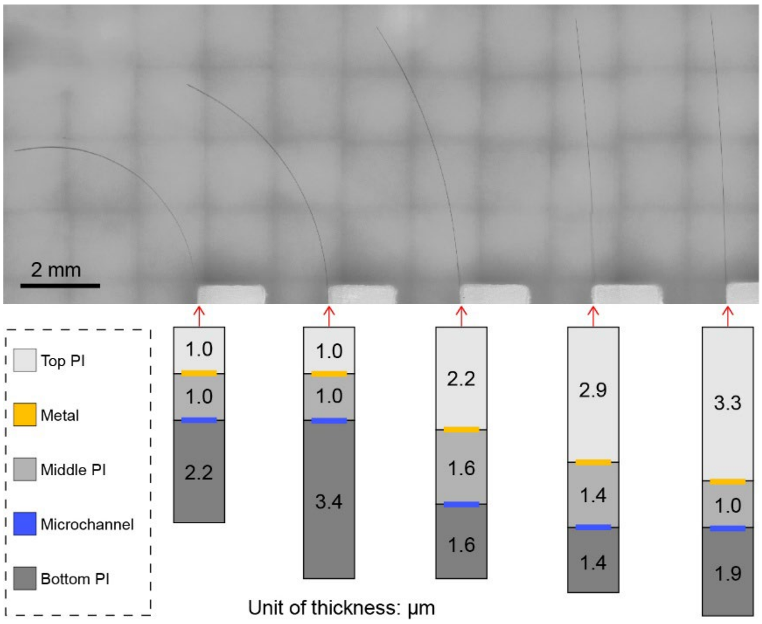

Figure S3. Schematic diagram of opening the liquid inlet through the microstair

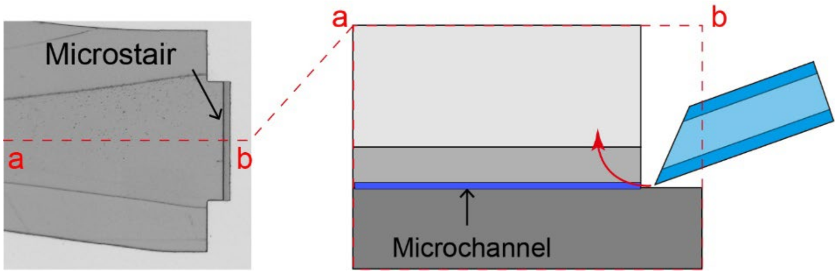

Figure S4. Procedures of encapsulating the neurotentacle with the liquid pathway and electrical connector

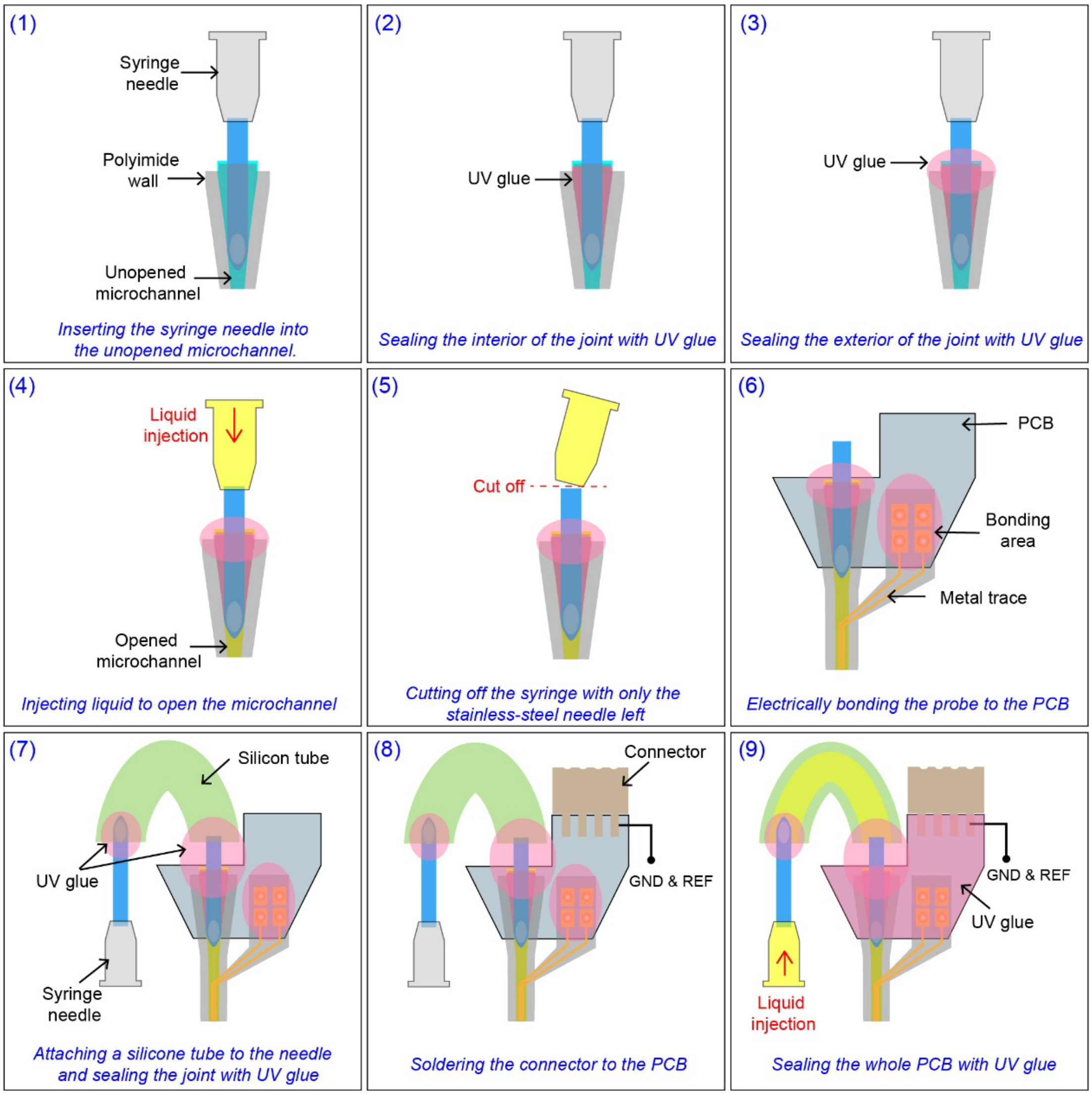

Figure S5. The relationship between loaded force (F_L) and downward displacement (Z) for neurotentacles under different pressures and the control tungsten wire (logarithmic scale on the x-axis)

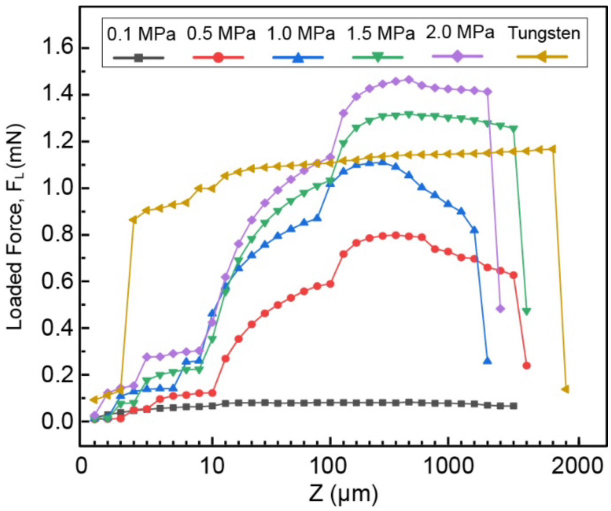

Figure S6. The relationships of average F_{MAX} and pressure (n = 5) for another three neurotentacles

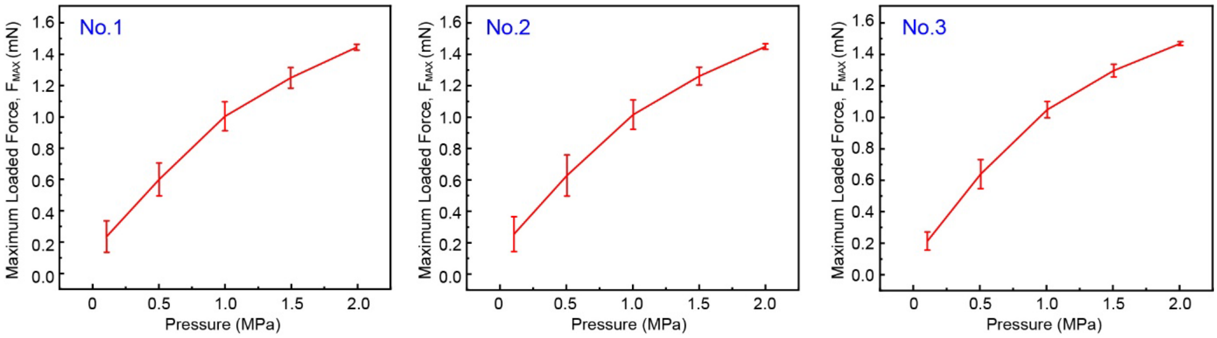

Figure S7. Bending resistance characterization of the Neurotentacle. **(A)** Schematic illustration of the testing conditions for radial and axial bending resistance. **(B)** Relationship between bending angle of the probe and pressure under radial loading. **(C)** Measurement procedure for axial deviation angle under radial loading. **(D)** Relationship between bending angle of the probe and pressure under axial loading. **(E)** Measurement procedure for axial deviation angle under axial loading.

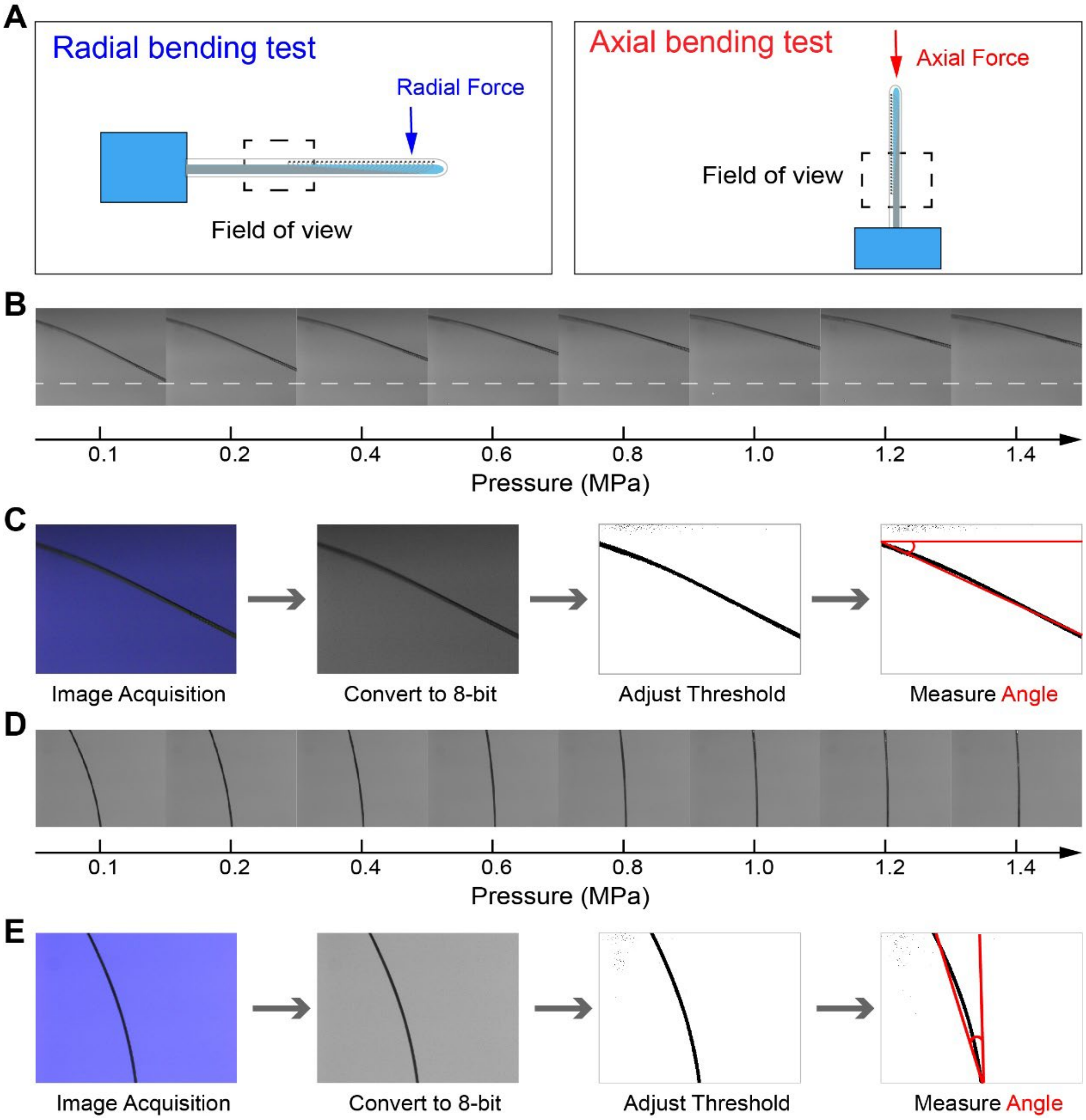

Figure S8. Experimental setup for testing the critical pressure of neurotentacles on the gel

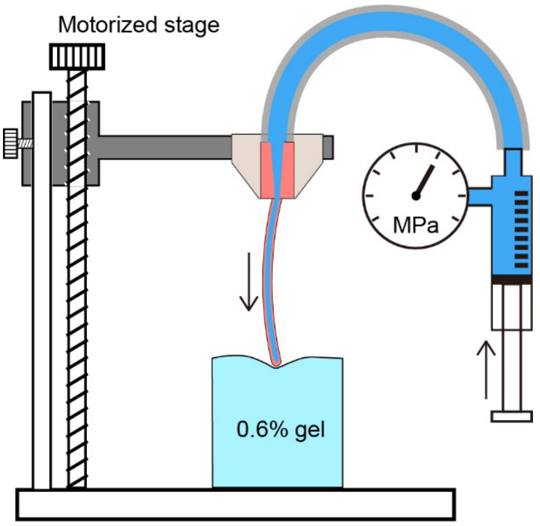

Figure S9. Experimental setup for testing the critical force needed for neurotentacles to penetrate the gel

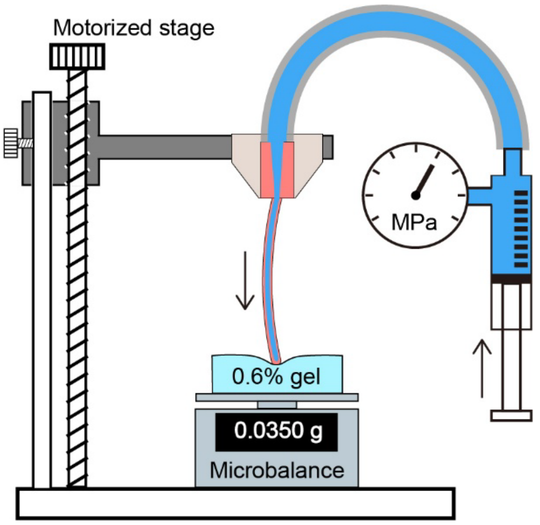

Figure S10. Procedures for acquiring cross-sections of neurotentacles under different pressures (1MPa for example) based on the flip-mould of PDMS

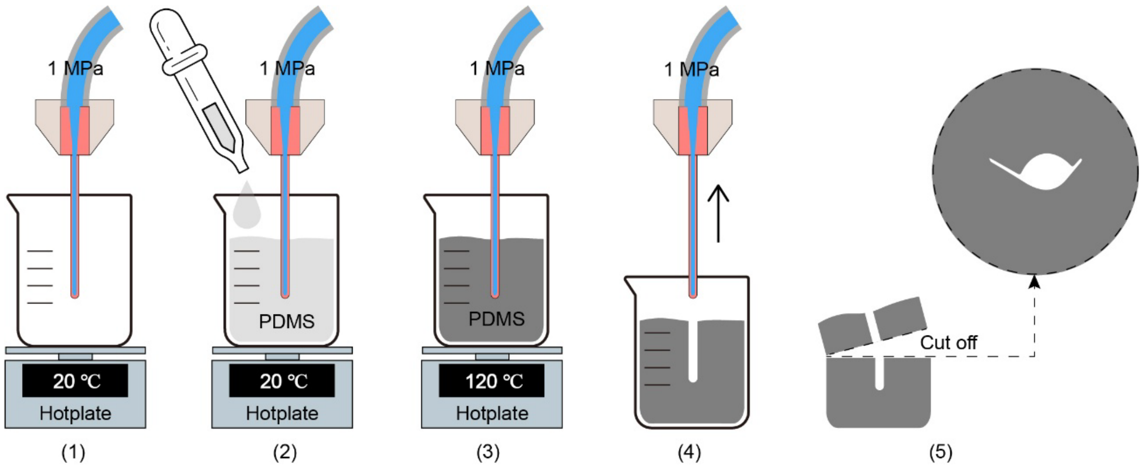

Figure S11. Morphological changes of the Neurotentacle during the full hydraulic actuation cycle

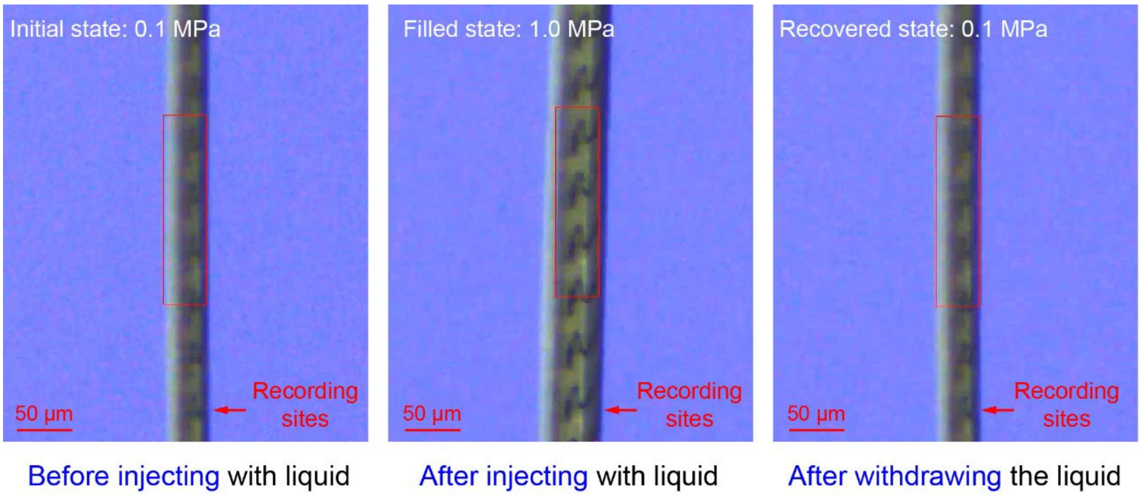

Figure S12. Layout design and prototype of the 32-channel neurotentacle probe

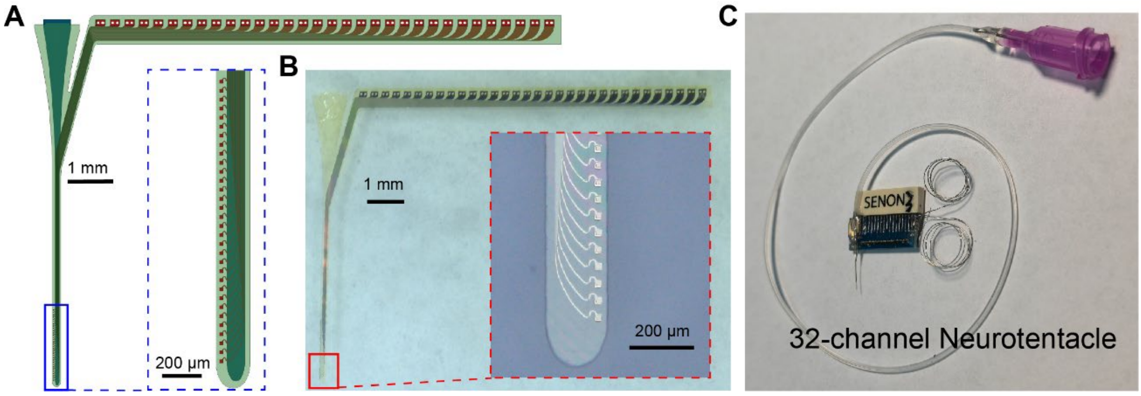

Figure S13. Neurotentacle probe with a hole at the tip for tungsten needle-assisted implantation. **(A)** Neurotentacle with a small hole of 30-40 μm in diameter at the tip. **(B)** T-shaped tungsten needle with a 100 μm diameter and a tip that is 20-30 μm in diameter. **(C)** Assembly of the probe and tungsten needle (100 μm). **(D)** The tip of the assembly.

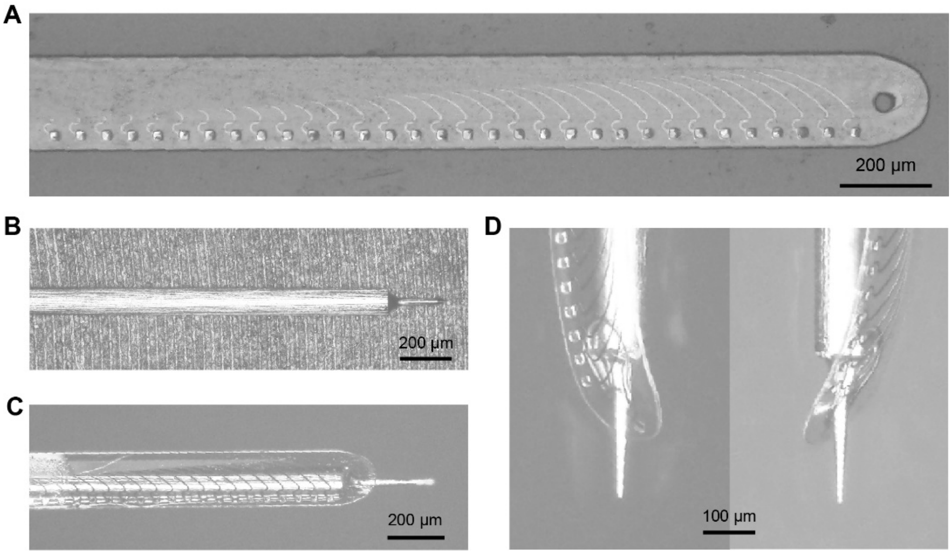

Figure S14. A 3D-printed needle-assisted probe insertion tool. **(A)** 3D model diagram of the implantation tool. **(B)** Assembled implantation tool with a flexible probe attached to it. **(C)** Implantation of a needle-assisted flexible probe in a mouse using the insertion tool. **(D)** Diagram illustrating the implantation process of the needle-assisted probe.

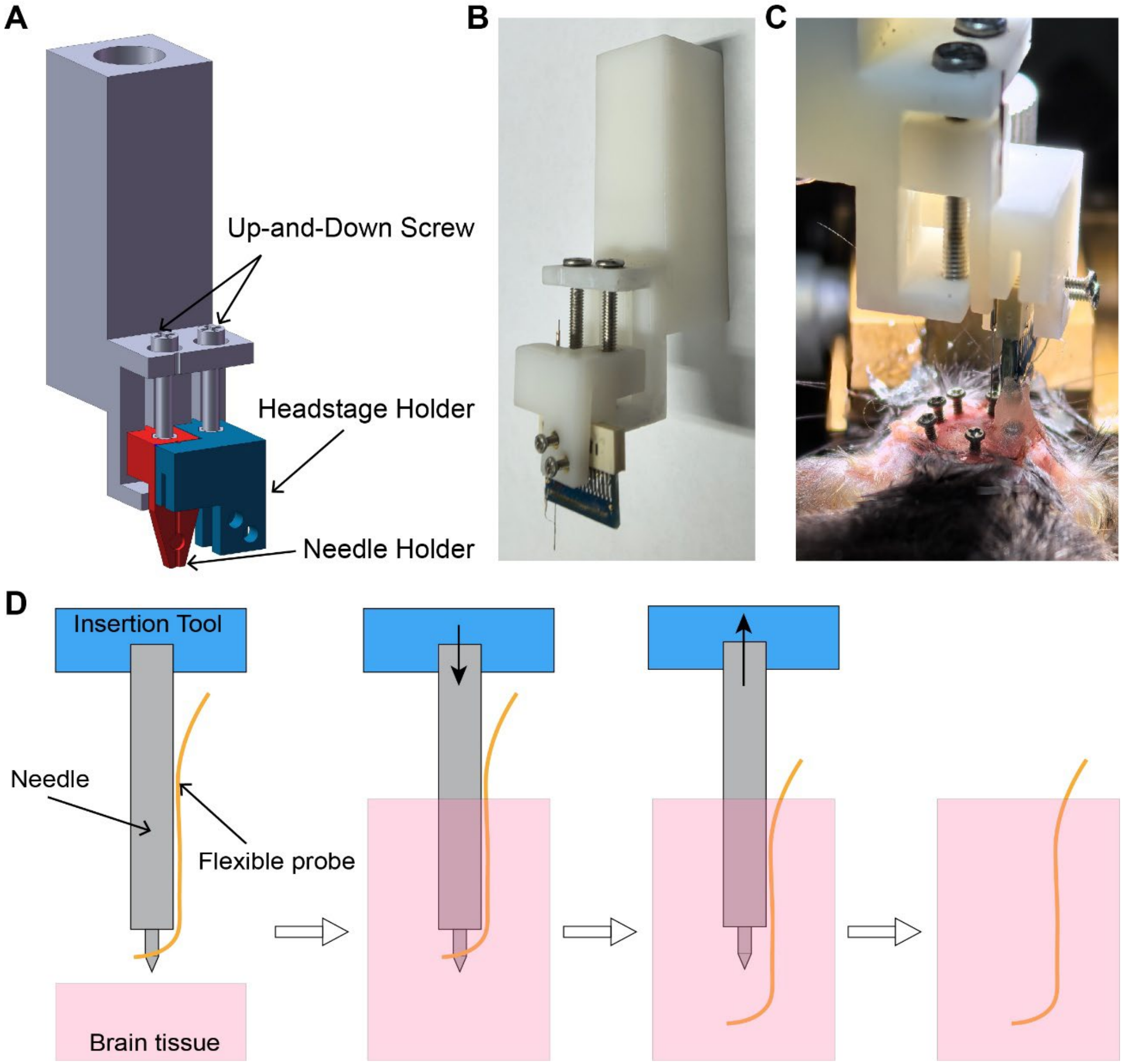

Figure S15. Simultaneous 64-channel electrophysiological recording in mice (left: hydraulic-based probe, Ch.01 - Ch.32; right: needle-assisted probe, Ch.33 - Ch.64)

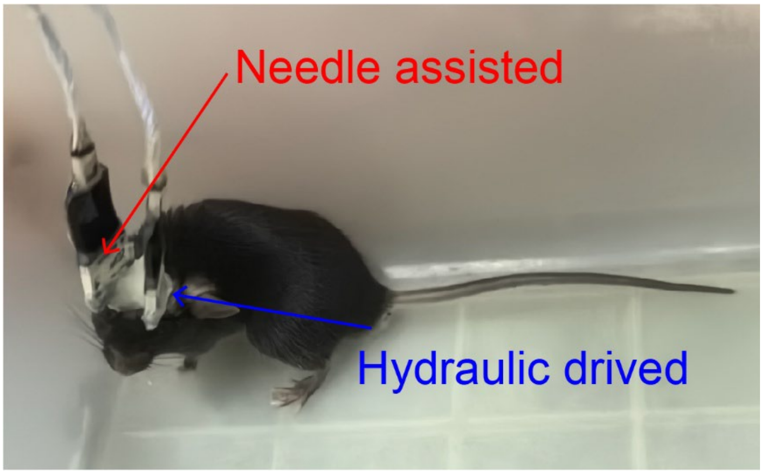

Figure S16. Acute brain sections (horizontal) from mouse #14 showing hydraulic-based probe (left) and 50 µm needle-assisted probe (right) (green for NeuN, blue for DAPI).

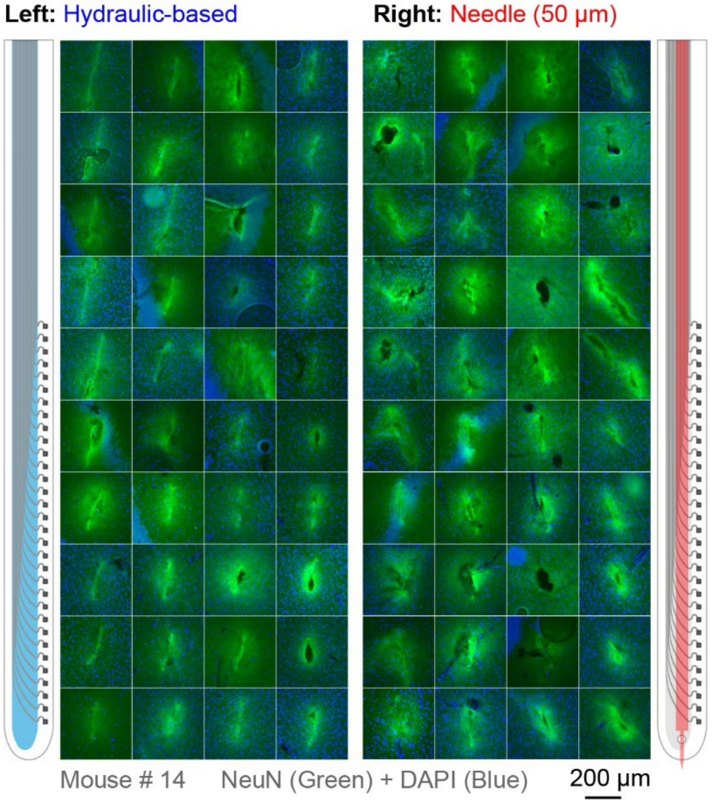

Figure S17. Steps for quantifying acute damage using ImageJ software

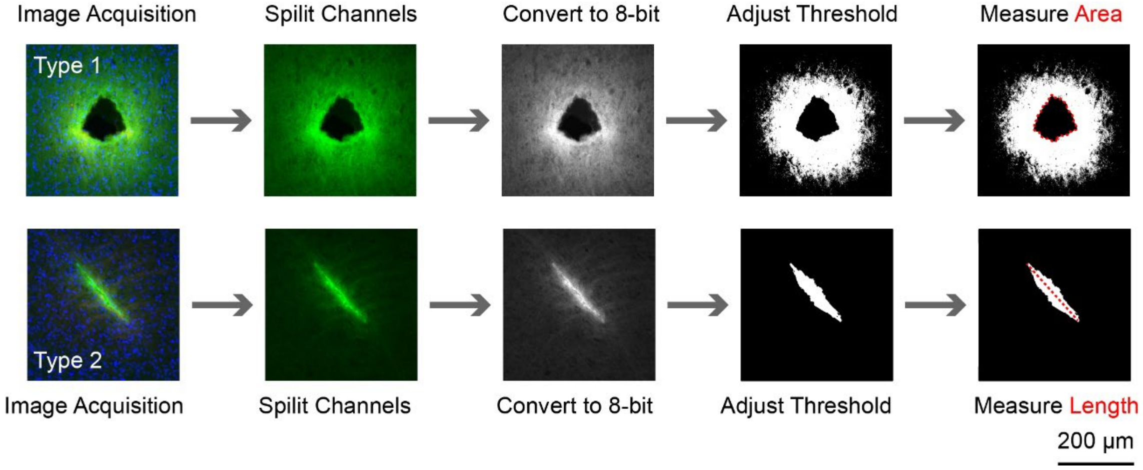

Figure S18. Chronic brain sections (horizontal) from mouse #7 after one month, showing hydraulic-based probe (left) and 100 µm needle-assisted probe (right) (purple for GFAP, blue for DAPI).

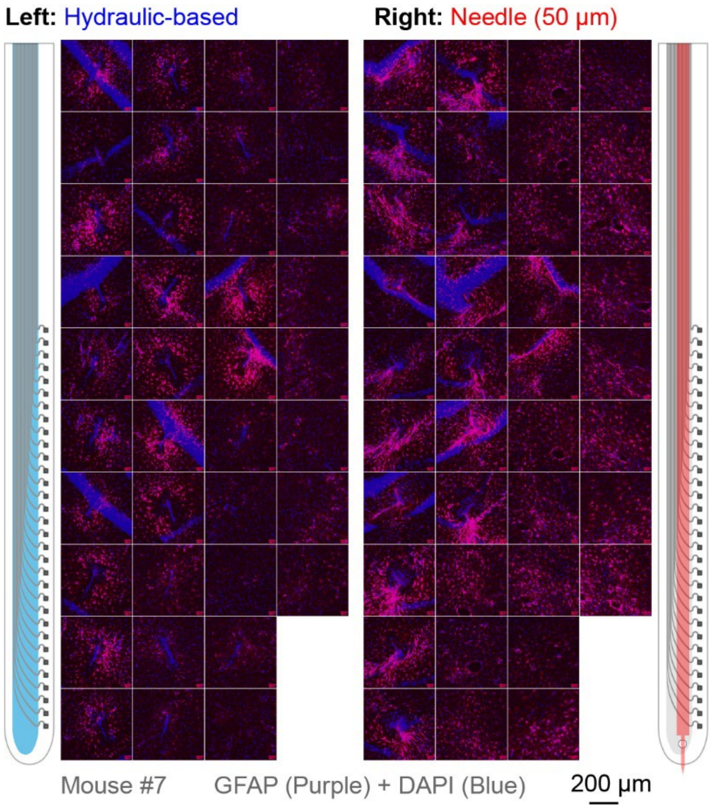

Figure S19. Steps for quantifying chronic immune response using ImageJ software

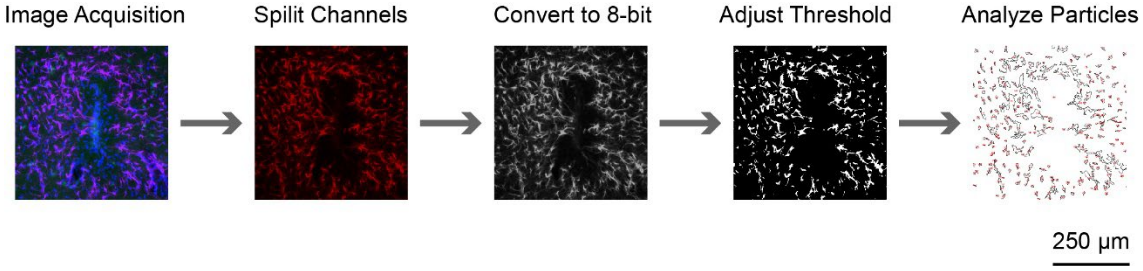

Figure S20. The relationship among buckling force (F_B), maximum loaded force (F_{MAX}), and insertion force (F_{IN}), and their relevance to insertion outcome. **(A)** The relationship based on typical loaded force – displacement curve. **(B)** The relationship based on the loaded force – displacement curve of the Neurotentacle.

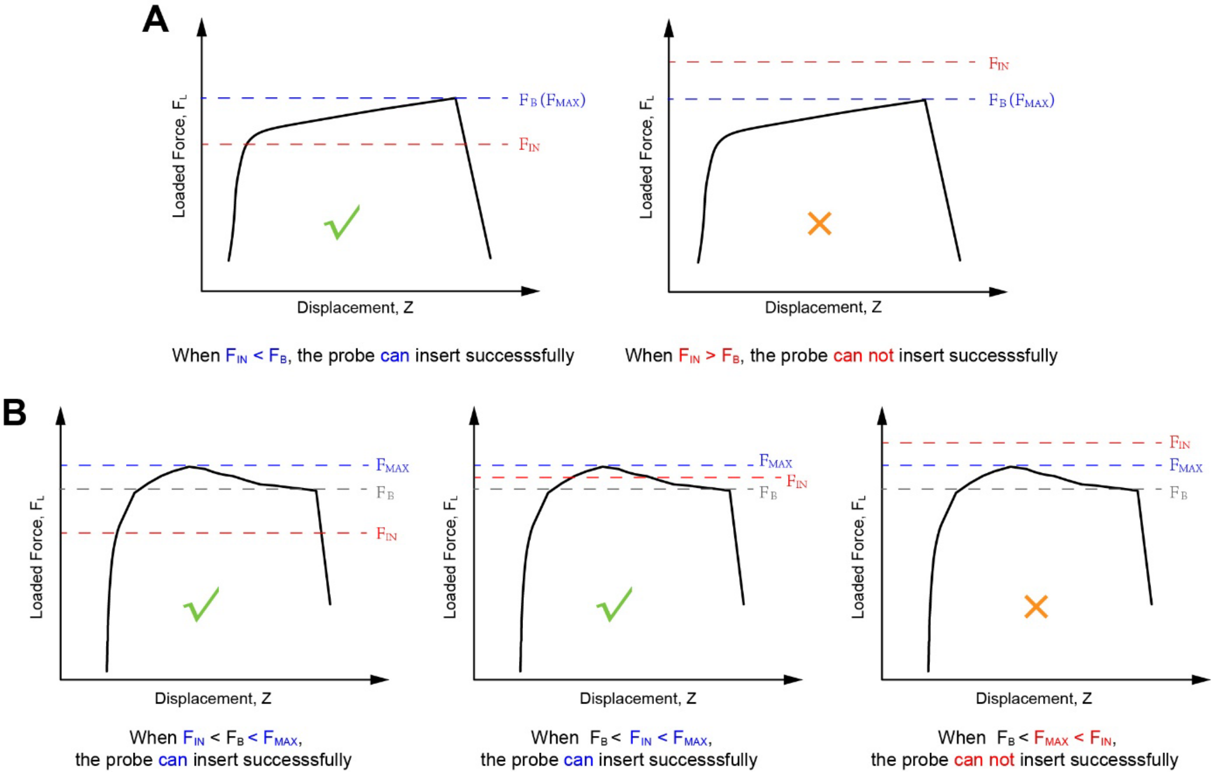

Figure S21. Insert the neurotentacles into the brain-like gel with varied pressure

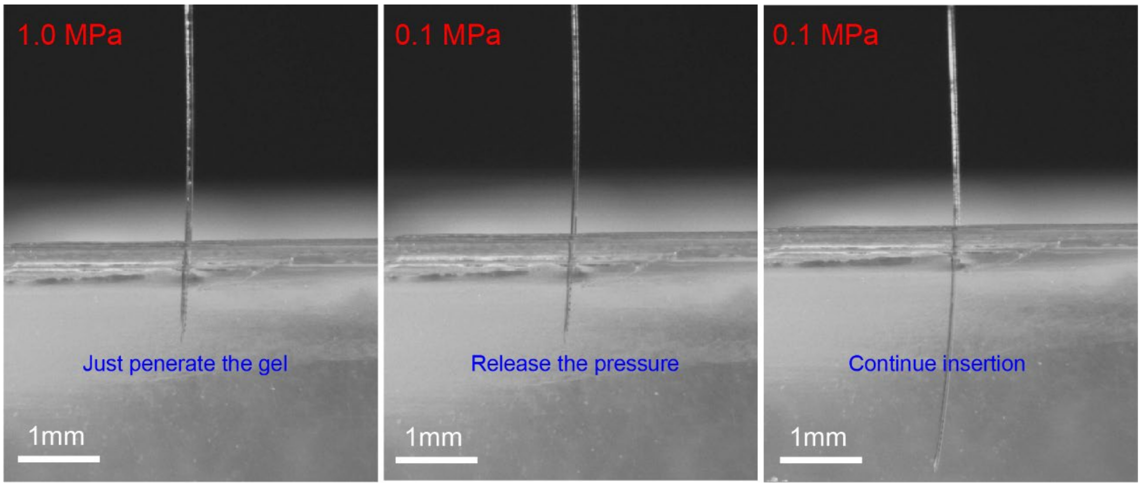

Figure S22. Simplified 2D finite element models in COMSOL Multiphysics for simulating the mechanical behavior of the Neurotentacle. **(A)** Cross-sectional model for structural deformation under different hydraulic pressures. **(B)** Longitudinal section model for axial stiffness variation under different hydraulic pressures.

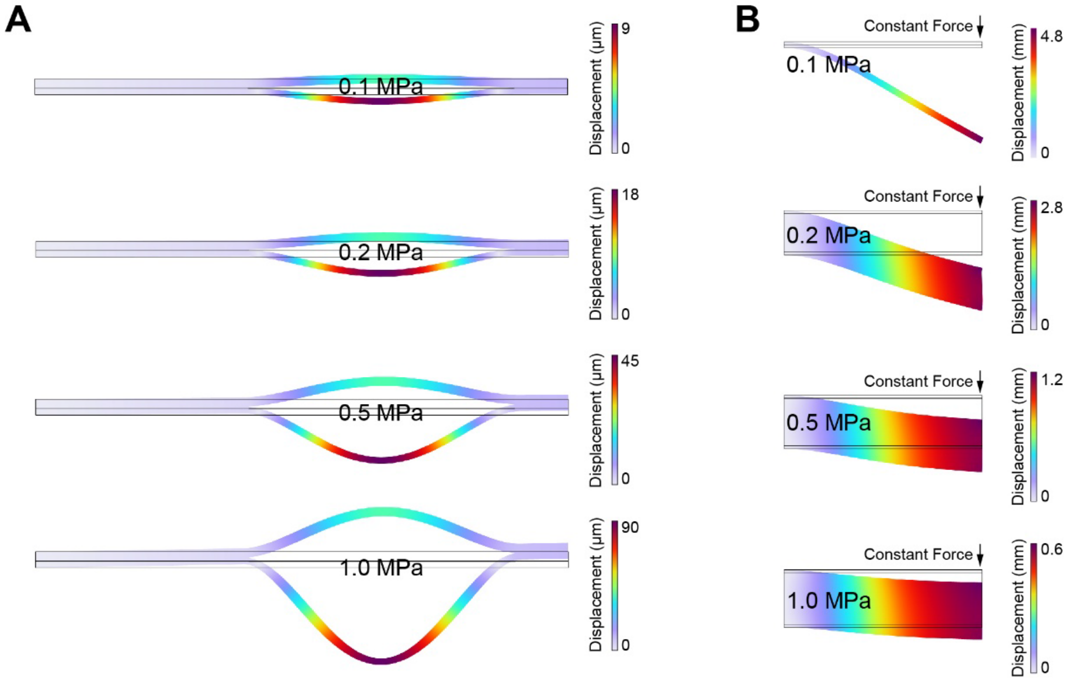

Table S1. Uncalibrated cross-sectional parameters of a neurotentacle

Pressure (MPa)	Width (μm)	Thickness (μm)	Area (μm^2)	Perimeter (μm)
**0.0**	182.4	9.9	1404.0	370.0
**0.1**	192.2	17.6	1961.2	394.6
**0.2**	162	45.6	3391.4	372.8
**0.5**	154.9	54.4	4053.2	375.7
**1.0**	147.9	58.6	4014.8	372.6

Table S2. Calibrated cross-sectional parameters of a neurotentacle

Pressure (MPa)	Width (μm)	Thickness (μm)	Area (μm^2)	Perimeter (μm)
**0.0**	182.4	9.9	1404.0	370.0
**0.1**	180.2	16.5	1724.3	370.0
**0.2**	160.8	45.3	3340.6	370.0
**0.5**	152.5	53.6	3931.1	370.0
**1.0**	146.9	58.2	3959.0	370.0

Table S3. Characteristic dimensions of the shuttles used in the literature

Shuttle Materials	Probe Width (μm)	Shuttle Dimension (μm^2)	Author
**Tungsten**	350	**Φ 140-175**	Richter et al., 2013
**Silicon**	250	**220×50**	Felix et al., 2013
**Tungsten**	8	**Φ 50**	Zhao et al., 2019
**Tungsten**	50	**Φ 40**	musk et al., 2019
**Tungsten**	100	**Φ 50**	Yasar et al., 2023
**Tungsten**	-	**Φ 100**	Liu et al., 2024
**SU-8**	300	**300×40**	Zhao et al., 2023

Movie S1. Implantation process of the neurotentacle

Movie S2. Bending angle response of the Neurotentacle under a constant load at the tip

Movie S3. Critical implantation pressure testing of neurotentacles on the brain-like gel

Movie S4. Dynamic morphological response of the Neurotentacle to internal pressure

Movie S5. Inserting neurotentacles with variable stiffness in the brain-like gel
